# FaPAO5 regulates Spm/Spd levels as a signaling during strawberry fruit ripening

**DOI:** 10.1002/pld3.217

**Published:** 2020-04-29

**Authors:** Aowai Mo, Tian Xu, Qian Bai, Yaunyue Shen, Fan Gao, Jiaxuan Guo

**Affiliations:** ^1^ Beijing Collaborative Innovation Center for Eco‐Environmental Improvement with Forestry and Fruit Trees Beijing University of Agriculture Beijing China; ^2^ Bei Jing Bei Nong Enterprise Management Co., Ltd Beijing China

**Keywords:** ABA, FaPAO5, polyamine, strawberry fruit ripening

## Abstract

Polyamines are important for non‐climacteric fruit ripening according to an analysis of the model plant strawberry. However, the molecular mechanism underlying the polyamine accumulation during ripening has not been fully elucidated. In this study, an examination of our proteome data related to strawberry fruit ripening revealed a putative polyamine oxidase 5, FaPAO5, which was localized in the cytoplasm and nucleus. Additionally, *FaPAO5* expression levels as well as the abundance of the encoded protein continually decreased during ripening. Inhibiting *FaPAO5* expression by RNAi promoted Spd, Spm, and ABA accumulation while inhibited H_2_O_2_ production, which ultimately enhanced ripening as evidenced by the ripening‐related events and corresponding gene expression changes. The opposite effects were observed in *FaPAO5*‐overexpressing transgenic fruits. Analyses of the binding affinity and enzymatic activity of FaPAO5 with Spm, Spd, and Put uncovered a special role for FaPAO5 in the terminal catabolism of Spm and Spd, with a *K*_d_ of 0.21 and 0.29 µM, respectively. Moreover, *FaPAO*5 expression was inhibited by ABA and promoted by Spd and Spm. Furthermore, the RNA‐seq analysis of RNAi and control fruits via differentially expressed genes (DEGs) indicated the six most enriched pathways among the differentially expressed genes were related to sugar, abscisic acid, ethylene, auxin, gibberellin, and Ca^2+^. Among four putative *PAO* genes in the strawberry genome, only *FaPAO5* was confirmed to influence fruit ripening. In conclusion, FaPAO5 is a negative regulator of strawberry fruit ripening and modulates Spm/Spd levels as a signaling event, in which ABA plays a central role.

## 1 INTRODUCTION

Fruits, which are derived from flowers, finally develop to produce mature seeds and accessory tissues, including the pericarp, ovary, receptacle, and/or rachis. Ripened fruits are often juicy, sweet or sour, and edible, and are also abundant in sugars, vitamins, minerals, antioxidants, and fibers, which are essential components of the human diet and health. The fruit ripening process involves complex physiological, biochemical, and molecular changes that influence fruit textures, colors, flavors, and aromas. On the basis of a sharp increase in the respiration rate and ethylene emission, the ripening of fleshy fruits is divided into climacteric and non‐climacteric processes. There has been considerable progress in the functional characterization of ethylene in climacteric fruits, such as tomato (*Solanum lycopersicum*; Alexander & Grierson, 2002; Liu, Pirrello, Chervin, Roustan, & Bouzayen, 2015), and the elucidation of the roles of ABA in non‐climacteric fruits, including strawberry (*Fragaria ananassa*; Li, Jia, Chai, & Shen, 2011; Rose & Shen, 2014). Notably, there is increasing evidence that polyamines (PAs) substantially affect fruit ripening (Kushad, Yelenosky, & Knight, 1988; Dibble, Davies, & Mutschler, 1988; Rodriguez, López, & Chaves, 1999; Lester, 2000; de Dios, Matilla, & Gallardo, 2006; Parra‐Lobato et al., 2011; Koushesh, Arzani, & Barzegar, 2012; Gupta, Pal, & Rajam, 2013; Jiang et al., 2015; Fortes, Teixeira, & Agudelo‐Romero, 2015; Simpson et al., 2017; Guo et al., 2018; Liu et al., 2018). Although PAs are important for strawberry fruit ripening, the molecular mechanisms underlying PA accumulation remain unclear.

It is now accepted that plant polyamines, including Put, Spd, and Spm, take part in various biological processes, such as morphogenesis, senescence, and fruit development, as well as abiotic and biotic stresses (Handa & Mattoo, 2010; Teh et al., 2014; Minocha, Majumdar, & Minocha, 2014; Ebeed, Hassan, & Aljarani, 2017; Guo et al., 2018; Fortes & Agudelo‐Romero 2018; Handa, Fatima, & Mattoo, 2018; Hao et al., 2018; Romero et al., 2018; Zahedi, Hosseini, Karimi, & Ebrahimzadeh, 2019). Since early reports on a role of PAs in fleshy fruits (Cohen, Arad, Heimer, & Mizrahi, 1982; Winer, Vinkler, & Apelbaum, 1984), over the past years, much progress has been made toward understanding PA roles in fruit development and ripening (Kushad et al., 1988; Dibble et al., 1988; Rodriguez et al., 1999; Lester, 2000; Bregoli et al., 2002; de Dios et al., 2006; Parra‐Lobato & Gomez, 2011; Koushesh et al., 2012; Kitashiba, Hao, Honda, & Moriguchi, 2005; Jiang et al., 2015; Fortes et al., 2015; Simpson et al., 2017; Guo et al., 2018). Taken together, the effects of PAs vary with the composition and content of polyamines, fruit types, and developmental stages, to a certain extent, the ratios of (Spd + Spm)/Put control ripening of climacteric fruits (Guo et al., 2018; Liu et al., 2018).

The known fruit ripening‐related tomato mutants have enabled researchers to use tomato as a model species for studying the effects of PA on ripening (Dibble et al., 1988; Gapper, McQuinn, & Giovannoni, 2013; Hao et al., 2018; Liu et al., 2018; Mattoo et al., 2006, 2007; Mehta et al., 2002; Mutschler, 1984; Saftner & Baldi, 1990; Sharma, Pareek, Sagar, Valero, & Serrano, 2017; Tassoni, Watkins, & Davies, 2006; Tsaniklidis et al., 2016). An increase in Put contents may prolong the tomato fruit ripening process (Dibble et al., 1988; Saftner & Baldi, 1990; Tassoni et al., 2006; Tsaniklidis et al., 2016). In transgenic tomato plants, the overexpression of the yeast S‐adenosylmethionine decarboxylase (SAMDC) gene (*ySAMdc*) that functions in the production of decarboxylated S‐adenosylmethionine (dcSAM) for both Spd and Spm biosynthesis, results in the ripening‐specific accumulation of Spd, Spm, and ethylene, which promotes the lycopene accumulation and quality of fruits (Mehta et al., 2002). Analyses of the *ySAMdc*‐overexpressing (OE) transgenic tomato plants and metabolic activities revealed that PAs function as anti‐apoptotic regulatory molecules to revive tomato fruit metabolic memory (Mattoo et al., 2006, 2007). Although PA biosynthesis in tomato fruits is committed to Arg or Orn, the induction of the Orn pathway decreases in the later ripening stages (Lasanajak et al., 2014). In transgenic tomato fruits expressing the mouse ornithine decarboxylase (*ODC*) gene, the Put, Spd, and Spm contents reportedly increase, while ethylene production, the respiration rate, and water loss decline, ultimately resulting in a considerably delayed fruit ripening (Pandey, Gupta, Chowdhary, Pal, & Rajam, 2015). Spatial and temporal analyses of PA biosynthesis and the expression of metabolism‐related genes, including *ODC*, *ADC* (arginine decarboxylase), *CuAO* (copper‐containing amine oxidase, also known as *DAO*), *SPDS* (Spd synthase), *SPMS* (Spm synthase), *SAMDC*, and *PAO* (polyamine oxidase), indicated that these genes more or less take part in PA biosynthesis and metabolism during the early fruit development stage. Moreover, the high *CuAO* and *SPMS* transcription levels during the later fruit development stage are consistent with a sharp increase in fruit size. Furthermore, *SPDS1* is the most highly expressed of these genes during the fruit ripening process, and most of these genes are highly expressed in fast‐growing tissues, with *CuAO* playing an especially important role in ripening fruits (Tsaniklidis et al., 2016). A recent study proved that the *SlPAO2*, *3*, and *4* expression levels are upregulated, whereas *SlPAO1*, *5*, *6*, and* 7* expression levels are downregulated in developing tomato fruits (Hao et al., 2018).

Grape and strawberry are typically used for investigating non‐climacteric fruit ripening. Compared with the climacteric fruit ripening involving ethylene, non‐climacteric fruit ripening is an ethylene‐independent process with only slight changes in ethylene emission (Alexander & Grierson, 2002). Although Put and Spd contents sharply decrease, while Spm is maintained at stable levels during grape berry ripening (Shiozaki, Ogata, & Horiuchi, 2000), PA catabolism indeed plays a vital role in berry enlargement and aroma production (Agudelo‐Romero et al., 2014). The *OsPAO5* transcript level gradually increases during rice seed germination, but this increase is inhibited by 5 mM guazatine (Chen et al., 2016). An increase in PAO‐derived hydrogen peroxide (H_2_O_2_) levels is terminated by the addition of the PAO‐specific inhibitor guazatine in Arabidopsis (Toumi et al., 2019). Exogenous ABA enhances the expression of the maize polyamine oxidase gene, which contributes to the ABA‐induced cytosolic antioxidant defense via H_2_O_2_ (Xue, Zhang, & Jiang, 2009).

Regarding strawberry, substantial progress has been made in elucidating the roles for PA in non‐climacteric fruit ripening. Specifically, Spm contents increase sharply after the onset of coloration, relative to the Put and Spd contents, causing Spm to become the dominant component in ripened fruits. Additionally, exogenous Put retards ripening, while exogenous Spm and Spd treatments have the opposite effect. Manipulating *FaSAMDC* expression alters fruit ripening, and the encoded enzyme is highly active (*K*_d_ of 170 µM), demonstrating that Spm and FaSAMDC are vital for strawberry fruit ripening (Guo et al., 2018). In comparison to more progress in the role PAs in climacteric tomato fruit ripening, a role of PAs in non‐climacteric fruit ripening is just onset (Guo et al., 2018), many important issues remain to be solved, especially a role of PA metabolism in strawberry fruit ripening.

In the present study, on the basis of our proteome data, a probable polyamine oxidase 5, FaPAO5, was screened, after which a similar trend in the transcript and protein levels during ripening was confirmed. Pharmacological, physiological, biochemical, and molecular analyses were completed, including a subcellular localization as well as an examination of expression‐influencing elements, transient transgenic fruits, and RNAi (RNA interference) and transgenic fruit RNA‐seq data, in addition to an evaluation of enzymatic activities based on HPLC and isothermal titration calorimetry (ITC). We determined that FaPAO5 negatively modulates strawberry fruit ripening by regulating Spm/Spd levels.

## 2 MATERIALS AND METHODS

### 2.1 Plant material

Strawberry (*Fragaria* × *ananassa* cultivar "Zhangji") plants were cultivated in a greenhouse at 23–28°C with 60%–70% relative humidity in the spring of 2017 and 2018. On the basis of a previous report (Jia et al., 2011), the following seven strawberry fruit developmental stages were analyzed: SG, LG, DG, Wt, IR, PR, and FR, which corresponded to 7, 13, 17, 22, 24, 26, and 28 DAA, respectively. Twenty fruits of a uniform size were sampled at each stage (*n* = 20; each fruit representing a biological replicate) and then immediately frozen in liquid nitrogen and stored at − 80°C until analyzed.

### 2.2 Proteome analysis of strawberry fruit ripening

A combination of tandem mass tag quantitative proteomics and liquid chromatography tandem mass spectrometry analyses were completed by Jingjie PTM Biolab Co. Ltd. (Hangzhou, China). Specifically, strawberry fruits from the LG, Wt, IR, and PR stages were examined. The extracted proteins were separated by 12% sodium dodecyl sulfate polyacrylamide gel electrophoresis to ensure the quality of the extractions was appropriate. Protein concentrations were determined with a BCA kit (Biyuntian, China). Differentially abundant proteins were defined as those with abundances that were >2‐fold (*p* < .05) different among fruit development stages. Proteins were functionally classified based on Gene Ontology terms from the following three main categories: biological process, cellular component, and molecular function. Additionally, the enriched KEGG pathways (http://www.genome.jp/kegg/) among the differentially abundant proteins were identified based on a two‐tailed Fisher's exact test. The GO/KEGG annotations with a corrected p‐value < 0.05 were considered significant. This analysis was repeated three times. The mass spectrometry proteomics data have been deposited to the ProteomeXchange Consortium via the PRIDE partner repository with the dataset identifier PXD018073.

### 2.3 Subcellular localization

The *FaPAO5* coding sequence was cloned into the pSuper1300‐GFP vector at the *Spe*I and *Kpn*I restriction enzyme sites (Primers used to amplify *FaPAO* and vector construction see Table S3). The FaPAO5‐GFP recombinant plasmid was inserted into tobacco (*N. tabacum*) leaf cells via an *A.* *tumefaciens* (strain GV3101) mediated infiltration. At 72 hr after the infiltration, the inferior tobacco leaf epidermis was removed and the GFP signal was observed with the LSM 710 META confocal laser‐scanning microscope (Zeiss, Germany). The analysis was repeated three times.

### 2.4 Determination of PA, anthocyanin, and soluble sugar contents

Three randomly selected fruits were analyzed by HPLC to quantify the PA, anthocyanin, and soluble sugar contents as previously described (Guo et al., 2018). The HLPC was completed with the following columns: ZORBAX Eclipse XDB‐C18 column (4.6 × 250 mm, 5 µm; Agilent) at 30°C for detecting PAs; the ZORBAX Eclipse XDB‐C18 column (4.6 × 150 mm, 5 µm; Agilent) for detecting anthocyanins; and the Agilent Technologies 1,200 Series Sugar‐Pak™ column (6.5 × 300 mm; Waters, Milford, MA, USA) for detecting soluble sugars. The analysis was repeated three times.

### 2.5 Determination of fruit firmness and soluble solid concentrations

Three fruits were evaluated to determine the fruit firmness and soluble solid concentrations. Fruit firmness was measured with the FHM‐5 fruit hardness tester (Takemura Electric Works Ltd, Tokyo, Japan). The soluble solid concentrations in receptacles were determined with the MASTER‐100H sugar analysis instrument (ATAGO, Tokyo, Japan) as described by Jia et al. (2013). The analyses were repeated three times.

### 2.6 RNA isolation, cDNA synthesis, gene cloning, and qPCR

Total RNA was extracted from 3 g receptacle tissue from three randomly selected fruits with the OMEGA RNA extraction kit (OMEGA Biotek, Norcross, GA, USA). The purity and integrity of the RNA were assessed by gel electrophoresis and A260/A230 and A260/A280 ratios. The RNA (0.4 µg) was used as the template to synthesize the first‐strand cDNA with the Trans kit (TransGen, Beijing, China).

A qPCR assay was performed with SYBR® Green (TransGen) and the LightCycler® 96 Real‐Time PCR System (Roche Diagnostics GmbH, Mannheim, Germany) as previously described (Huang, Xu, Hou, & Shen, 2019). *Actin* was used as a reference gene (Jia et al., 2013; Wang et al., 2017). Relative gene expression levels were analyzed according to the 2^−ΔΔCt^ method (Livak & Schmittgen, 2001). Details regarding the primers used for the qPCR assay and *FaPAO5* cloning are listed in Tables S4 and S5. The qPCR analysis was repeated three times.

### 2.7 Construction of RNAi and OE plasmids and fruit agroinfiltration

The Gateway system was used for constructing the RNAi and OE plasmids carrying the strawberry transgene. Specifically, the pDONR™ 221(entry vector), pK7GWIWG2(II)RR (RNAi), and pH7FWG‐RR (OE) vectors were used. Additionally, 436‐bp and 1,659‐bp *FaPAO5* cDNA fragments were cloned to construct the RNAi and OE recombinant plasmids, respectively. The recombinant plasmids were transferred into *E. coli* strain trans1‐T1 (TransGen). Details regarding the primers used for the qPCR assay and *FaPAO5* cloning are listed in Tables S2 and S3. *Agrobacterium tumefaciens* (strain GV3101) carrying the FaPAO5_436_ *‐*pK7GWIWG2‐RNAi and FaPAO5_1659_‐pH7FWG‐RR‐OE recombinant plasmids were used for the infiltration of 20 DG strawberry fruits. Specifically, the fruits were injected with 100–200 µl *A. tumefaciens* suspensions with a hypodermic syringe as previously described (Guo et al., 2018).

### 2.8 In vitro fruit disk incubation

Three randomly selected white strawberry fruits were cut into 1‐mm disks. The fruit disks were treated with 100 µM Put, Spd, Spm, ABA, guazatine, or fluridone. The basic treatment solution (i.e., without these compounds) was used as a control. Samples were incubated for 2 hr as described by Jia et al. (2013). A qPCR analysis of *FaPAO5* expression was completed as described above. The analysis was repeated three times.

### 2.9 Isothermal titration calorimetry assay

The final concentration of the recombinant FaPAO5 fusion protein was adjusted to 10 µM in ITC buffer (200 mM NaCl, 20 mM Tris, 0.5 mM EDTA, and 10 mM maltose, pH 7.4). The ITC analysis was conducted with 10 µM recombinant FaPAO5 and 100 µM ligands (Put, Spd, and Spm; Sigma‐Aldrich) in an ITC200 calorimeter (MicroCal, Northampton, MA, USA) at 30°C. The protein samples were titrated with a 2‐μL injection of ligands every 5 min as described by Wild et al. (2016). Data were fitted with the ORIGIN 7.0 software (MicroCal). The assay was repeated three times.

### 2.10 HPLC analysis of FaPAO5 activity and its products

To analyze the products of PA oxidation as described by Wang & Liu (2016), 10 μg purified FaPAO5 was incubated with 150 mM Spd in 100 mM phosphate buffer (pH 7.5) at 38°C or with 150 mM Spm in 100 mM phosphate buffer (pH 6.5) at 36°C. Samples were analyzed at different time‐points. The reaction products were extracted with precooled perchlorate (5% volume fraction), which was followed by the derivation and benzoylation of the extracted PAs and Dap. The substrates and products were examined by HPLC with the ZORBAX Eclipse XDB‐C18 column (4.6 × 250 mm, 5 μm; Agilent) at 30°C. The mobile phase was methanol:water (v:v, 64:36) for 20 min at a flow rate of 0.7 ml/min. The effluent absorbance was monitored at 230 nm. The injection volume was 10 ml. The HPLC experiment was repeated three times.

### 2.11 RNA‐sequencing of RNAi and control fruits

To further clarify the negative regulatory function of FaPAO5 during strawberry fruit ripening, the RNA‐seq analysis of *FaPAO5*‐RNAi and control fruits was performed. Three fruits were randomly sampled for an RNA isolation and cDNA synthesis, which were completed as previously described (Wang et al., 2017). Total RNA was extracted from receptacles of RNAi and control fruits with the RNeasy Plant Mini kit (Qiagen, Dusseldorf, Germany). The RNA was treated with the RNase‐Free DNase set (Qiagen) to eliminate any contaminating DNA. The RNA was then used to construct libraries with the RNA Library Prep kit (New England BioLabs, Ipswich, MA, USA) for a subsequent sequencing with the Illumina HiSeq 2000 platform (BioProject accession number in NCBI: PRJNA612621). Unigenes were annotated based on available databases, including the NCBI non‐redundant protein (NR), COG, KEGG, and GO databases. The adjusted *p*‐value < .05 and |log_2_ fold‐change| ≥ 1 were used to evaluate DEGs. The analysis was repeated three times.

## 3 RESULTS

### 3.1 Expression pattern and subcellular localization of FaPAO5 based on proteomic data

An analysis of our proteome data for large green (LG), white (Wt), initially red (IR), and partially red (PR) fruits around the onset of strawberry fruit ripening uncovered three polypeptides related to a putative polyamine oxidase 5 in the National Center of Biotechnology Information (NCBI) database (https://blast.ncbi.nlm.nih.gov/Blast.cgi) that continually decreased in abundance (log2 fold‐change ≥ 1) (Fig. S1a). The screened protein, which was named FaPAO5, comprised 552 amino acids and was encoded by a 1,659‐bp coding sequence. In developing fruits, a quantitative real‐time PCR (qPCR) analysis indicated that the *FaPAO5* expression level was continually downregulated from the small green (SG) to the LG, de‐greening (DG), Wt, IR, and PR fruit stages, but increased slightly from the PR to FR fruit stages (Fig. S1b). These results suggested that FaPAO5 might negatively regulate fruit ripening.

**Figure 1:**
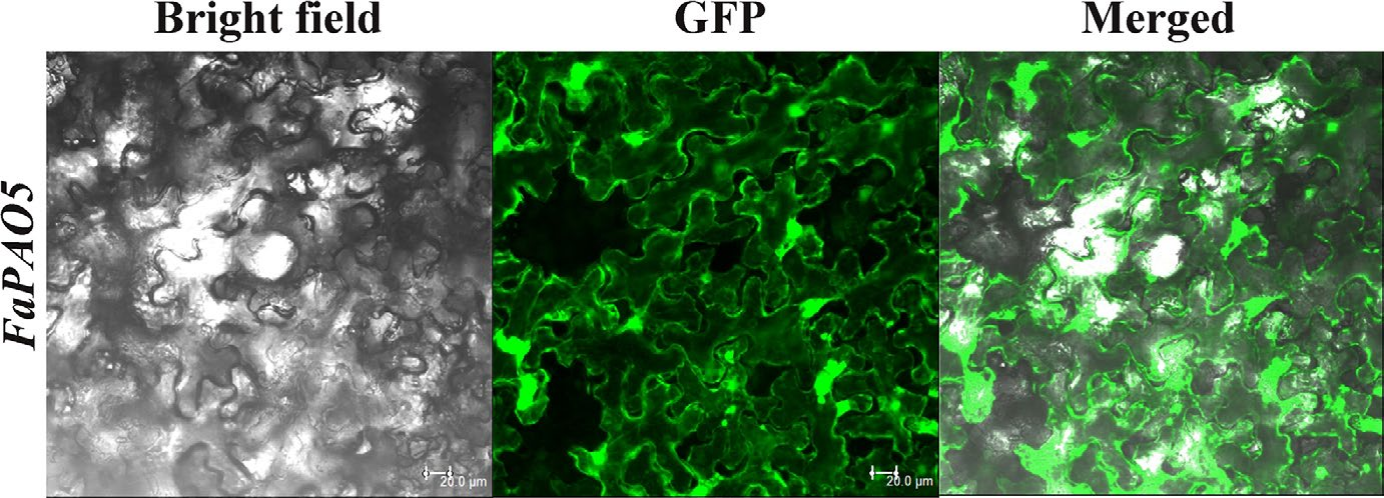
Subcellular localization of FaPAO5. Localization analysis was performed to use a green fluorescent protein (GFP)‐pCAMBIA1300‐ProSuper–FaPAO5 fusion protein (GFP‐FaPAO5) in *Nicotiana tabacum* leaves; and the GFP:pCAMBIA1300‐ProSuper (1300‐GFP) was used as control. Fluorescence images were obtained by confocal laser‐scanning microscope. The nuclei stained by 4′,6′‐diamidino‐2‐phenylindole (DAPI) appear in blue. Scale bar = 20 μm

The Plant‐PLoc computational results indicated FaPAO5 may be localized in the Cytoplasm. To confirm this prediction, the pCAMBIA1300‐FaPAO5‐GFP (GFP‐FaPAO5) subcellular localization vector was used for the transient transformation of tobacco (*Nicotiana tabacum*) leaf cells, after which the GFP signal was observed by fluorescence microscopy, with control leaves transformed with pCAMBIA1300‐GFP (1300‐GFP). By DAPI staining the fusion protein signal was localized in the Cytoplasm and nucleus. The results demonstrated that FaPAO5 is localized in the Cytoplasm and nucleus (Figure 1).

### 3.2 Factors influencing *FaPAO5* expression detected in the fruit disk incubation test

**Figure 2:**
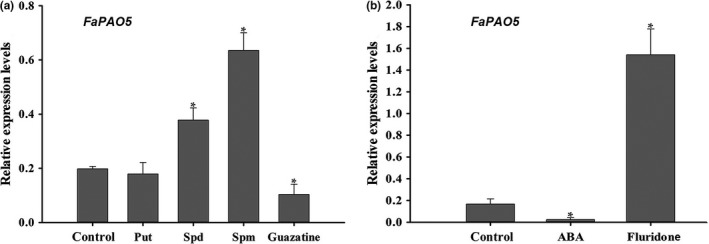
Factors influencing *FaPAO5* expression. Effects of 100 µM Put, Spd, Spm, ABA, guazatine, and fluridone on *FaPAO5* expression were investigated via an in vitro white‐fruit disk incubation method. (a) Effects of Put, Spd, Spm, and guazatine on *FaPAO5* expression. (b) Effects of ABA and fluridone on *FaPAO5* expression. The *actin* expression level was used as an internal control. Bars represent standard errors (*n* = 3). The asterisk in the columns indicates significant differences (*p* < .05) compared with the control value according to an ANOVA followed by Duncan's multiple range test

To explore the effects of Spm, Spd, ABA, guazatine, and fluridone on *FaPAO5* expression, fruit disk and pharmacological tests were performed. The *FaPAO*5 expression level increased in response to Spd, Spm, and fluridone (an ABA biosynthesis inhibitor), decreased after an exposure to ABA and guazatine, and was unaffected by Put (Figure 2a and b). These results indicated that ABA and guazatine inhibit *FaPAO*5 expression, whereas Spd and Spm have the opposite effect. Therefore, *FaPAO5* expression may be influenced by Spm/Spd/ABA, but not by Put. Accordingly, Spm/Spd may be a positive regulator, whereas ABA is a negative regulator for *FaPAO*5 expression.

### 3.3 Functional analysis of FaPAO5 in strawberry fruits

**Figure 3:**
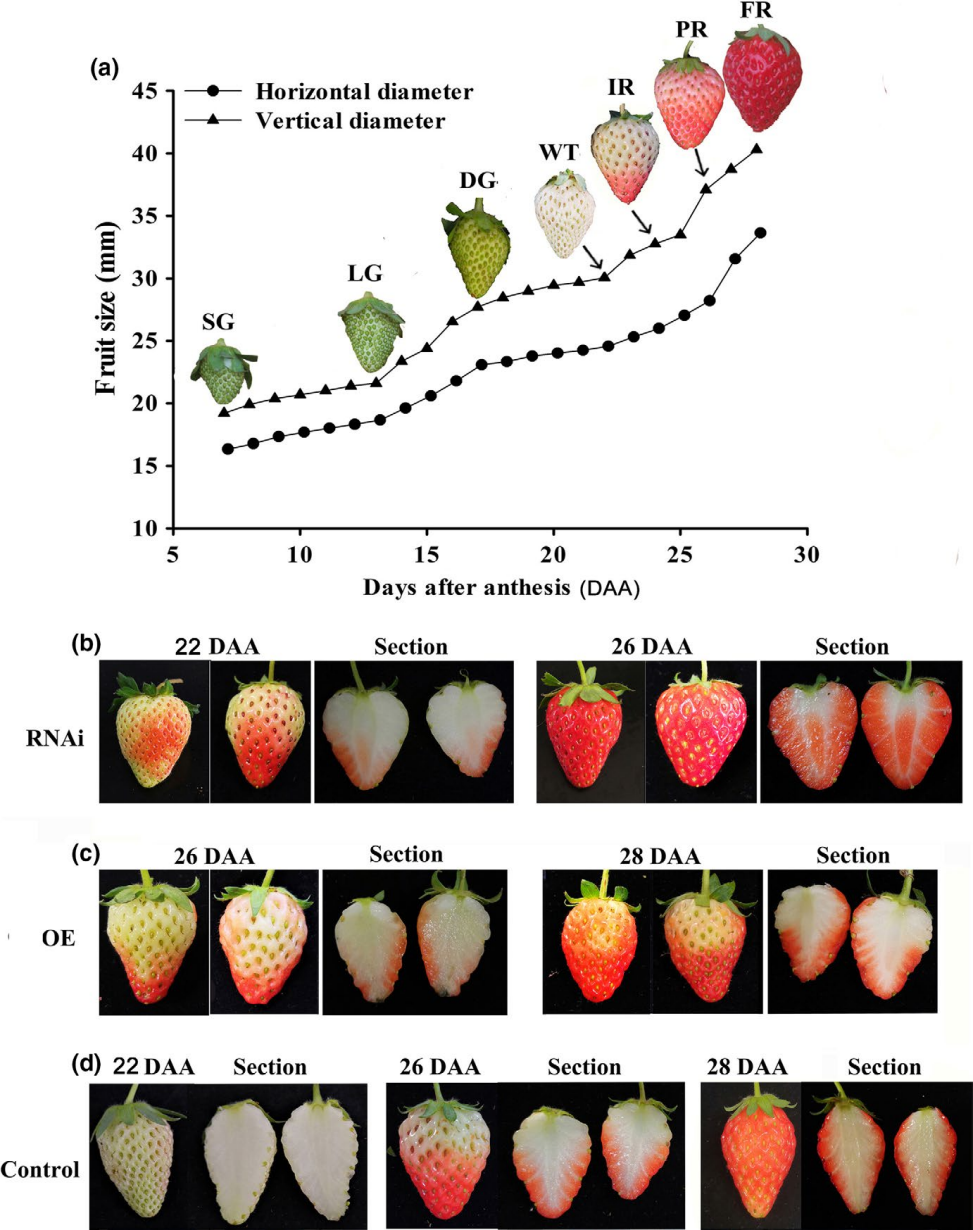
Phenotypes of wild‐type and *FaPAO5‐*overexpressing transgenic strawberry fruits. (a) The seven developmental stages were as follows: small green (SG), large green (LG), de‐greening (DG), white (Wt), initially red (IR), partially red (PR), and fully red (FR). (b) Phenotypes of the RNAi fruits were examined at 4, 8, and 10 days after injections or 22, 26, and 28 days after anthesis (DAA). (c and d) Phenotypes of the OE (c) and control (d) fruits were examined at 4, 8, and 10 days after injections or 22, 26, and 28 DAA

In a previous study (Jia et al., 2011), the developmental stages for "Zhangji" (*Fragaria* × *ananassa*) fruits were divided into the SG, LG, DG, Wt, IR, PR, and fully red (FR) stages, which corresponded to 7, 13, 17, 22, 24, 26, and 28 days after anthesis (DAA), respectively (Figure 3a).

**Figure 4:**
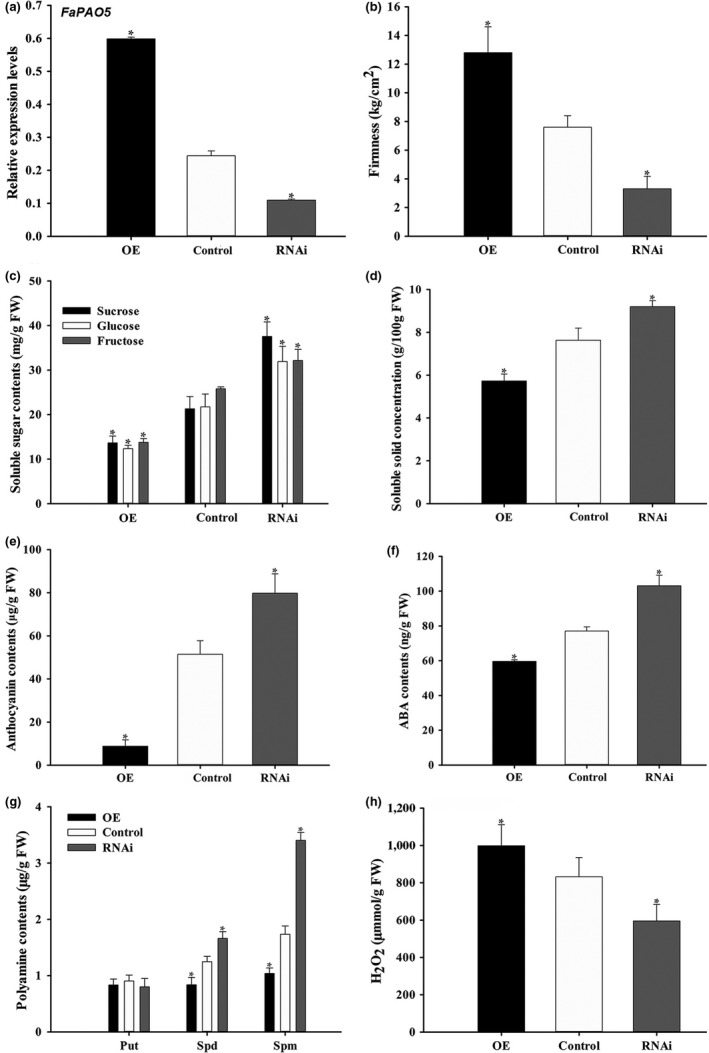
Manipulation of *FaPAO5* expression affects ripening‐related physiological parameters. (a) The *FaSAMDC* RNAi and OE fruits with significantly downregulated (RNAi) and upregulated (OE) expression levels relative to the control levels were analyzed. (b) Fruit firmness. (c) Soluble sugar contents. (d) Soluble solid concentrations. (e) Anthocyanin contents. (f) ABA levels. (g) Polyamine contents. (b) H_2_O_2_ production. The *actin* expression level was used as an internal control. Bars represent standard errors (*n* = 3). The asterisk in the columns indicates significant differences (*p* < .05) compared with the control value according to an ANOVA followed by Duncan's multiple range test

To confirm that FaPAO5 is a negative regulator of strawberry fruit ripening, *Agrobacterium* tumefaciens (strain GV3101) cells containing RNAi [intron–hairpin RNA interference via pK7GWIWG2(II)RR‐*FaPAO_436_*] or FaPAO5‐OE (overexpression via pH7FWG‐RR‐*FaPAO_1659_*) recombinant plasmids or empty vectors (control) prepared with the Gateway construction system (Wang et al., 2018) were separately injected into 20 DG fruits on plants at 18 DAA. The fruit phenotypes were examined at 4, 8, and 10 days after injection or at 22, 26, and 28 DAA. The results indicated that downregulated *FaPAO5* expression promoted the development of red fruits at the IR (4 days/22 DAA) and FR (8 days/26 DAA) stages (Figure 3b). In contrast, upregulated *FaPAO5* expression inhibited the development of red fruits, and resulted in chimeric coloration at 8/10 days and 26/28 DAA (Figure 3c), unlike the control fruits (Figure 3d). Finally, the ratios of dark red: red: chimeric fruits in the RNAi, OE, and control groups were 18:2:0, 0:1:19, and 3:17:0, respectively. The qPCR data confirmed that the *FaPAO5* transcription levels were significantly downregulated by an average of 57% and upregulated by an average of 156% in the RNAi and OE fruits, respectively, relative to the control levels (Figure 4a). These data indicated that FaPAO5 serves as a negative regulator of strawberry fruit ripening.

### 3.4 Manipulation of *FaPAO5* expression affects ripening‐related processes at physiological and molecular levels

To characterize the role of FaPAO5 in the regulation of strawberry fruit ripening, the transgenic dark red RNAi and chimeric OE fruits (Figure 3) were used to analyze a set of ripening‐related parameters at physiological and molecular levels, including fruit firmness, soluble solid concentrations, soluble sugar contents (glucose, fructose, and sucrose), and anthocyanin contents. We also examined the PA, H_2_O_2_, and ABA levels and the expression of the genes encoding the following proteins: cinnamate‐4‐hydroxylase (C4H), 4‐coumarate:CoA ligase 1 (4CL1), chalcone synthase (CHS), chalcone isomerase (CHI), flavanone 3‐hydroxylase (F3H), dihydroflavonol 4‐reductase (DFR), flavonoid 3‐O‐glucosyltransferase (UFGT), anthocyanidin synthase (ANS), pentaerythritol (PE), pectate lyase (PL), polygalacturonase 1 (PG), carboxyl ester lipase (CEL), alpha‐xylosidase 1 (XYL1), expansin 2 (EXP2), Suc transporter 1 (SUT1), sucrose synthase (SS), SNF1‐related protein kinase 2.6 (SnRK2.6), 9‐cis‐epoxycarotenoid dioxygenase 1 (NCED1), protein phosphatase 2C37 (ABI37), ODC, ADC, SAMDC, SPDS, and SPMS (Guo et al., 2018; Jia et al., 2013, 2017).

**Figure 5:**
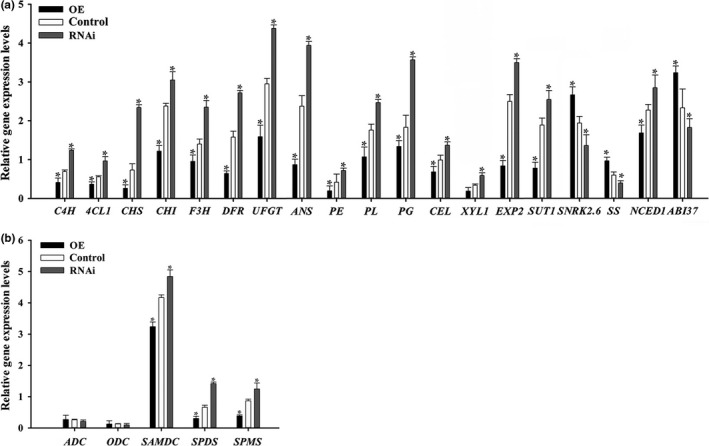
Manipulation of *FaPAO5* expression affects the expression of numerous ripening‐related genes. The *FaSAMDC* RNAi and OE fruits with significantly downregulated (RNAi) and upregulated (OE) expression levels relative to the control levels were analyzed. (a) Changes in the expression of genes related to pigmentation (*C4H*, *4CL1*, *CHS*, *CHI*, *F3H*, *DFR*, *UFGT*, and *ANS*), firmness (*PE*, *PL*, *PG*, *CEL*, *XYL1*, and *EXP2*), sugar contents (*FaSS* and *FaSUT1*), and ABA contents (*ABI37* and *SnRK2.6*). (b) Changes in the expression of genes related to polyamine biosynthesis (*ADC*, *ODC*, *SAMDC*, *SPDS*, and *SPMS*). The *actin* expression level was used as an internal control. Bars represent standard errors (*n* = 3). The asterisk in the columns indicates significant differences (*p* < .05) compared with the control value according to an ANOVA followed by Duncan's multiple range test

Fruit firmness decreased and increased in the RNAi and OE fruits, respectively (Figure 4b), relative to that of the control fruits (Figure 4). Compared with the corresponding levels in the control fruits, the soluble sugar (i.e., sucrose, glucose, and fructose) (Figure 4c), soluble solid (Figure 4d), anthocyanin (Figure 4e), and ABA (Figure 4f) contents were considerably lower in the OE fruits and higher in the RNAi fruits. The Spd and Spm contents decreased in the OE fruits and increased in the RNAi fruits, whereas the Put levels were unchanged relative to the levels in the control fruits (Figure 4g). The H_2_O_2_ levels decreased in the RNAi fruits, but increased in the OE fruits (Figure 3h). The qPCR assay revealed that in addition to the *SS* and *ABI37* expression levels, the transcript levels of *C4H*, *4CL1*, *CHS*, *CHI*, *F3H*, *DFR*, *UFGT*, *ANS*, *PE*, *PL*, *PG*, *CEL*, *XYL1*, *EXP2*, *SUT1*, *NCED1*, and *SnRK2.6* were significantly downregulated in the OE fruits and upregulated in the RNAi fruits relative to the control levels (Figure 5a). Notably, the *ADC* and *ODC* transcripts levels were not markedly affected in the transgenic fruits, whereas *SAMDC*, *SPDS*, and *SPMS* transcription was significantly downregulated in the OE fruits and upregulated in the RNAi fruit compared with the corresponding control levels (Figure 5b). Therefore, the changes in the expression of genes related to pigmentation (*C4H*, *4CL1*, *CHS*, *CHI*, *F3H*, *DFR*, *UFGT*, and *ANS*), firmness (*PE*, *PL*, *PG*, *CEL*, *XYL1*, and *EXP2*), sugar contents (*SS* and *SUT1*), ABA (*ABI37* and *SnRK2.6*), and PAs (*SAMDC*, *SPDS*, and *SPMS*) in the transgenic fruits provided insights into FaPAO5 as a negative regulator of fruit ripening.

### 3.5 Analysis of FaPAO5 enzymatic activity

**Figure 6:**
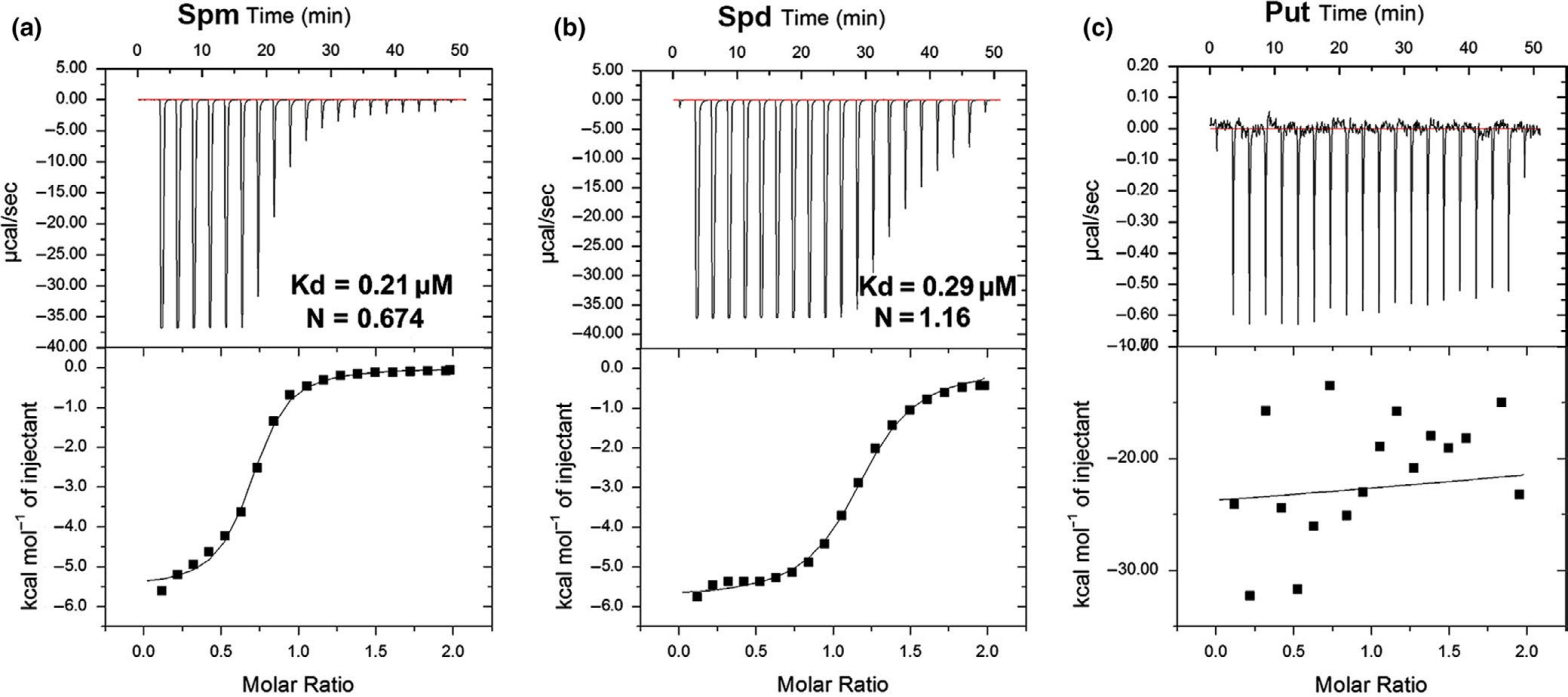
Affinity of the binding of FaPAO5 to polyamines based on isothermal titration calorimetry. (a) FaPAO5 exhibits strong Spm‐binding activity. (b) FaPAO5 exhibits high Spd‐binding activity. (c) FaPAO5 cannot bind Put

To investigate the FaPAO5 enzymatic activity, the corresponding coding sequence (from 1 to 1,659 bp) was expressed in *Escherichia coli* cells and purified. A 105‐kDa recombinant protein (1.5 mg/ml) was purified via MBP resin (Fig. S2a) and identified in a western blot with an anti‐His tag antibody (Fig. S2b). Additionally, an ITC titration was completed with 20 µM recombinant protein and 100 µM Put, Spd, or Spm, with a 2‐μL injection of PAs every 5 min. The data proved that the binding of FaPAO5 to Spm and Spd followed a saturation kinetics curve, with a dissociation constant (*K*_d_) of 0.21 µM (Figure 6a) and 0.29 µM (Figure 6b), respectively, and the stoichiometry (*N*) approached a 1:1 FaPAO5 to Spm/Spd‐binding ratio. However, there was no specific curve for the binding of FaPAO5 to Put (Figure 6c). These data demonstrated that FaPAO5 binds tightly to Spm/Spd, but not to Put.

**Figure 7:**
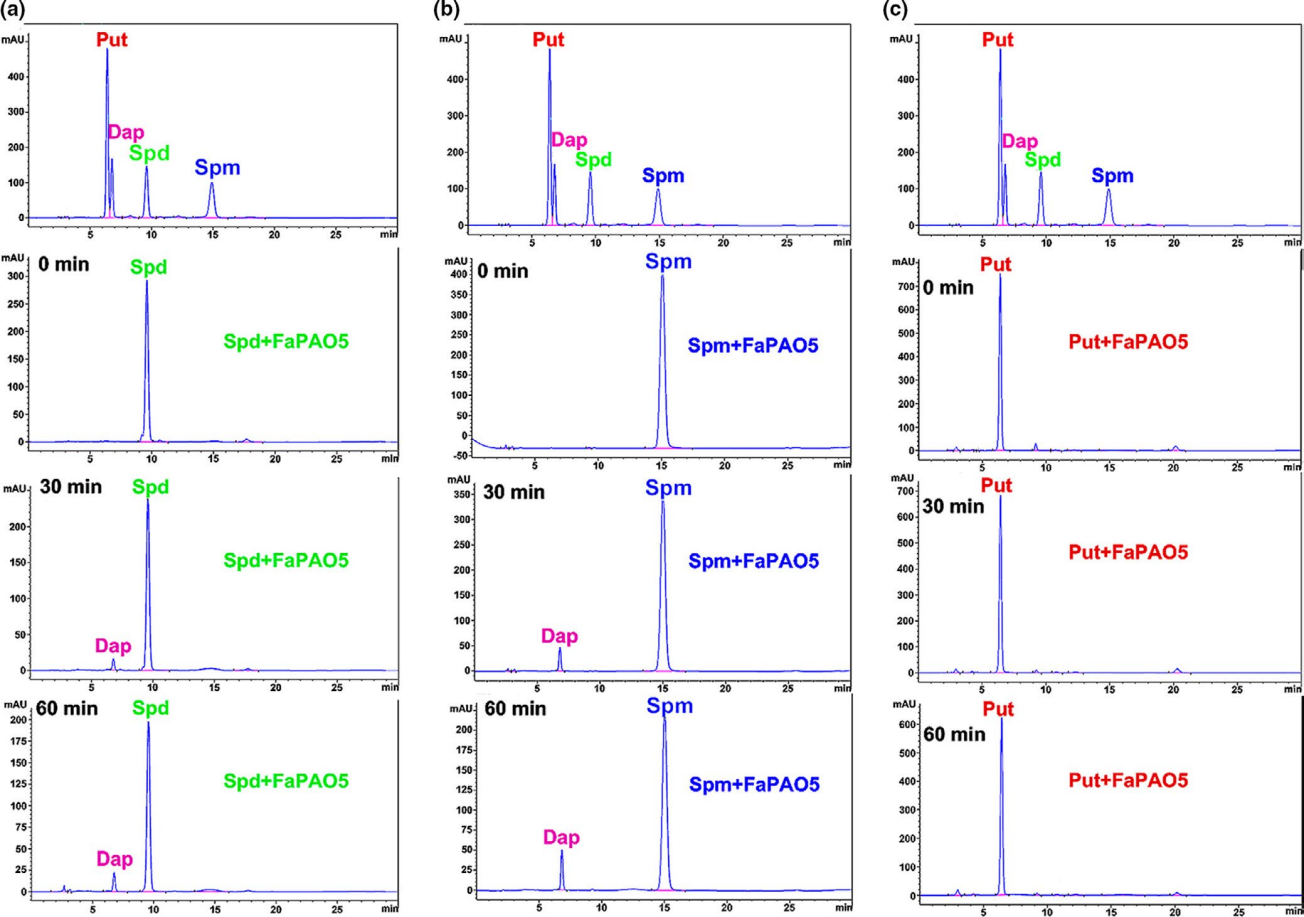
Metabolic analysis of FaPAO5 activity by HPLC. Polyamines (Spm, Spd, and Put) and a metabolic product [1,3‐diaminopropane (Dap)] were detected at 0, 30, and 60 min after initiating the polyamine–FaPAO5 reaction. (a) Spd + FaPAO5. (b) Spm + FaPAO5. (c) Put + FaPAO5

To further determine the enzymatic nature of FaPAO5, it was incubated with Spm/Spd/Put and the substrates and metabolic products were analyzed by HPLC. The HPLC results indicated that at the 0 min time‐point (i.e., before the reaction between FaPAO5 and the polyamines), only Spd/Spm/Put were detected. At 30 min after initiating the reaction, in addition to the substrates, 1,3‐diaminopropane (Dap) was detected as a product of the Spm/Spd reactions. The detected Dap signals increased at 60 min in a time‐dependent manner (Figure 7a and b). In contrast, Dap was undetectable in the Put reaction (Figure 7c). These results implied that FaPAO5 specifically degrades Spm/Spd via terminal metabolism (Cona, Rea, Angelini, Federico, & Tavladoraki, 2006). This observation was confirmed by the production of H_2_O_2_ in the reaction between FaPAO5 and Spm/Spd/Put at different pH and temperature conditions. Specifically, both pH and temperature affected H_2_O_2_ production, but not for Put (Fig. S3).

### 3.6 Transcriptome analysis of RNAi and control fruits

To clarify the negative regulatory function of FaPAO5 during strawberry fruit ripening, the RNAi and control fruits underwent an RNA‐seq analysis as previously described (Wang et al., 2017; BioProject accession number in NCBI: PRJNA612621). Data were obtained for 108,087 unigenes, of which 45,502 (42.1%) matched sequences in the NR database following Gene Ontology (GO) and COG analyses. The Kyoto Encyclopedia of Genes and Genomes (KEGG) pathway enrichment analysis resulted in 78,640 unigenes mapped to 383 pathways, with the five most enriched pathways associated with signal transduction (4,815 unigenes), infectious diseases (2,979 unigenes), translation (2,844 unigenes), carbohydrate metabolism (2,837 unigenes), and folding, sorting, and degradation (2,413 unigenes). A comparison of the DEGs between the RNAi and control fruits revealed 156 pathways associated with 912 unigenes. The five most enriched pathways were related to secondary metabolites, amino acid metabolism, carbohydrate metabolism, signal transduction, and lipid metabolism. The top six sub‐pathways were related to sugar (30 unigenes), ABA (21 unigenes), ethylene (11 unigenes), auxin (10 unigenes), gibberellin (10 unigenes), and Ca^2+^ (8 unigenes; Table S1). These unigenes were used as queries for a BLAST search and were annotated based on the transcriptome data for the LG, Wt, IR, and PR stages (Bioproject accession in GenBank: PRJNA438551; Guo et al., 2018). The role of FaPAO5 during strawberry ripening was clarified based on the set of abundant and potentially important transcripts, including those related to sugar (*MPG1*, *GUN8*, *SWET4*, *PGMP*, and *TPPA*), ABA (*PP2C37*, *CCD4*, and *NCED3*), ethylene (*EBF1*), IAA (*ARF6*, *IAA4*, and *ARP*), GA (*GASA6*), and Ca^2+^ (*MIRO1*, *ADT1*, and *CDPK4*; Table S2). These results suggested that sugar, phytohormones (especially ABA), and Ca^2+^ might be involved in the FaPAO5‐mediated regulation of strawberry fruit ripening.

### 3.7 Global analysis of the polyamine oxidase family

Plant PAOs generally belong to multiple gene families, with 11 members in the rice genome and at least five members in the Arabidopsis genome (Tavladoraki et al., 2006; Moschou, et al., 2008; Chen et al., 2016). To determine the functions of PAO family members during strawberry fruit ripening, we comprehensively examined our transcriptome data for the LG, Wt, IR, and PR fruit developmental stages (Bioproject accession in GenBank: PRJNA438551; Guo et al., 2018). We detected four *PAO* genes in the strawberry genome (*PAO1*, *PAO2*, *PAO4*, and *PAO5*). We observed that *PAO1* expression increased continuously, whereas *PAO4* and *PAO5* were stably expressed at low levels and *PAO2* expression tended to decrease (Fig. S4). On the basis of the subcellular localization and functional analysis of PAO5, we demonstrated that both PAO1 and PAO4 are located in the nucleus, whereas PAO2 is localized more in the nucleus rather than in the cytoplasm (Fig. S5). The downregulated expression of *PAO1*, *PAO2*, and *PAO4* did not result in observable phenotypic changes, implying these genes may not contribute to fruit ripening. Accordingly, among the strawberry *PAO* genes, *PAO5* appears to be critical for fruit ripening.

## 4 DISCUSSION

Although polyamines have relatively lower levels in the mature tomato fruits, indeed, PAs play a role in the ripening (Tsaniklidis et al., 2016). Actually, the roles of PAs show both short‐term and long‐term effects on peach fruit ripening (Torrigiani et al., 2012). In apple, Spd was a predominant form of polyamines during fruit development and ripening (Zhang et al., 2003). In eggplant fruit, dominant polyamines are Put and Spd, especially more Put, while no Spm found during ripening (Rodriguez et al., 1999). On the whole, the roles of PAs vary with their compositions and contents, fruit types, and developmental stages, to some extents, the ratios of (Spd + Spm)/Put control fruit ripening and quality (Guo et al., 2018).

It is previously reported that Arabidopsis AtPAOs mediates polyamine (PA) back‐conversion from Spm into Spd and then Put (Zarza et al., 2017). In the present study, we find a special role of FaPAO5 in degradation of Spm/Spd by the way of terminal metabolism rather that the back‐conversion metabolism, namely the purified FaPAO5 can specially oxidizes Spm or Spd into 1.3‐diaminopropane (Dap) and H_2_O_2_, and cannot play a role in the degradation of Put. Notably, the downregulation of *FaPAO5* expression decreased H_2_O_2_ levels while promoted ABA accumulation, finally accelerated ripening, thus we consider that ABA rather than H_2_O_2_ plays a vital role in the FaPAO5‐mediated ripening as a negative regulator. Given that ABA increases rapidly during strawberry fruit ripening (Jia et al., 2011), the higher ABA inhibited *FaPAO*5 expression, as a result, promoting both PA and ABA accumulation, thus a coordination mechanism between PA and ABA interaction, uncovers a new insight for the FaPAO5 in ripening, namely, FaPAO5, not only serves as enzyme function, but also acts as a signal role.

### 4.1 FaPAO5 plays a central role during strawberry fruit ripening

The rice *PAO* homologs are classified into four subfamilies (I, IIa, IIb, and III; Chen et al., 2016). The Arabidopsis genome encodes at least five putative PAOs, of which AtPAO1 converts Spm to Spd in a back‐conversion pathway and AtPAO3 catalyzes the sequential conversion of Spm to Spd and then to Put (Tavladoraki et al., 2006; Moschou et al., 2008). In the present study, we identified four putative *PAO* genes in the strawberry genome, among which *FaPAO1* and *FaPAO4* were classified into a large group that included *AtPAO2*, *AtPAO3*, and *AtPAO4*. Additionally, *FaPAO2* and *FaPAO5* were classified into another large group along with *OsPAO2* and *OsPAO5* (Fig. S6). Moreover, *FaPAO5* and *OsPAO5* were classified into a subgroup, whereas *AtPAO5* was included in another subgroup. Consistent with the subcellular localization of OsPAO5, both FaPAO2 and FaPAO5 were located in the nucleus and cytoplasm (Fig. S5), suggesting that FaPAO2 and FaPAO5 may influence gene expression and regulation. The fact that FaPAO1, 2, and 4 do not affect fruit ripening (data not shown) and only PAO5 is related to fruit ripening among the PAOs screened from the proteomic data imply that PAO5 is vital for regulating strawberry fruit ripening.

### 4.2 Polyamines play essential roles in strawberry fruit ripening

Over the past several decades, substantial progress has been made in characterizing the PA roles related to fruit development and ripening in tomato (Mutschler, 1984; Dibble et al., 1988; Saftner and Baldiet, 1990; Mehta et al., 2002; Tassoni et al., 2006; Mattoo et al., 2006, 2007; Gapper et al., 2013; Tsaniklidis et al., 2016; Sharma et al., 2017; Hao et al., 2018; Liu et al., 2018). Notably, a recent report described a positive role for Spm and Spd, especially Spm, in the regulation of strawberry fruit ripening because it manipulates the expression of *FaSAMDC*, which is important for the biosynthesis of Spd and Spm (Guo et al., 2018). This report provides an insight into the role of PAs in non‐climacteric fruit ripening via FaSAMDC. However, in contrast to the FaSAMDC‐mediated biosynthesis of Spd and Spm during ripening, a role for the PAO‐mediated degradation of Spd and Spm in ripening fruits remains unknown.

In the present study, proteomic data combined with the results of pharmacological, physiological, biochemical, and molecular analyses revealed that (a) FaPAO5 abundance and FaPAO5 expression continuously decrease during fruit development and ripening (Figure S1); (b) downregulated and upregulated *FaPAO5* expression induces and inhibits ripening, respectively (Figure 3); and (c) the *K*_d_ values of the reactions between FaPAO5 and Spm/Spd are 0.21/0.29 µM, respectively (Figure 6). These findings provide substantial evidence that FaPAO5 negatively influences fruit ripening, whereas Spm/Spd are positive regulators. Indeed, the results of a previous study on the FaSAMDC‐mediated biosynthesis of Spd and Spm to promote strawberry fruit ripening (Guo et al., 2018) and the data presented herein for the FaPAO5‐mediated metabolism of Spd and Spm to inhibit strawberry fruit ripening confirm that PAs (mainly Spm and Spd) play essential roles in strawberry fruit ripening. A coordinated regulation of PA contents by FaSAMDC and FaPAO5 contributes to the rapid accumulation of Spm and Spd, which induces ripening.

### 4.3 Characterizing the role of FaPAO5 in strawberry fruit ripening via ABA and Spm/Spd

Guo et al. (2018) reported that both FaSAMDC and Spm/Spd positively affect fruit ripening via an ABA‐dominated, IAA‐participating, and ethylene‐coordinated manner, implying a central role for the coordinated effects of ABA and PAs during ripening. In the present study, we determined that FaPAO5 negatively regulates strawberry fruit ripening, and inhibiting *FaPAO5* expression with guazatine promotes Spd and Spm accumulation, but does not affect Put contents, to accelerate ripening (Figure 2). The results of gene silencing and overexpression experiments indicated that *FaPAO5* does not affect the Put content, but actively alters the fruit firmness, H_2_O_2_ level, and *SS* and *ABI37* expression levels, while also negatively regulating most of the ripening‐related parameters at the physiological and molecular levels (Figures 4 and 5; Figures S7 and S8). These results imply that ABA, rather than H_2_O_2_, contributes to FaPAO5‐mediated activities during ripening. It is interesting that an increase in Spm/Spd contents resulting from a decrease in FaPAO5 expression may induce the *SAMDC*, *SPDS*, and *SPMS* expression levels (Figures 4 and 5; Figure S8b). These results indicate that a FaPAO5‐mediated increase in Spm/Spd levels may promote Spm/Spd biosynthesis‐related gene expression, thereby regulating the rapid accumulation of Spm and Spd. The fact that the binding of FaPAO5 to Spm or Spd separately reached a *K*_d_ of 0.21 or 0.29 µM (Figure 6), to some extent, suggests the binding of FaPAO5 to Spm/Spd serves as a signal that triggers *SAMDC*, *SPDS*, and *SPMS* expression to promote the rapid accumulation of Spm/Spd and ripening. Indeed, downregulated FaPAO5 expression may modulate the expression of genes related to ABA, ethylene, auxin, gibberellin, and Ca^2+^ (Table S1).

**Figure 8:**
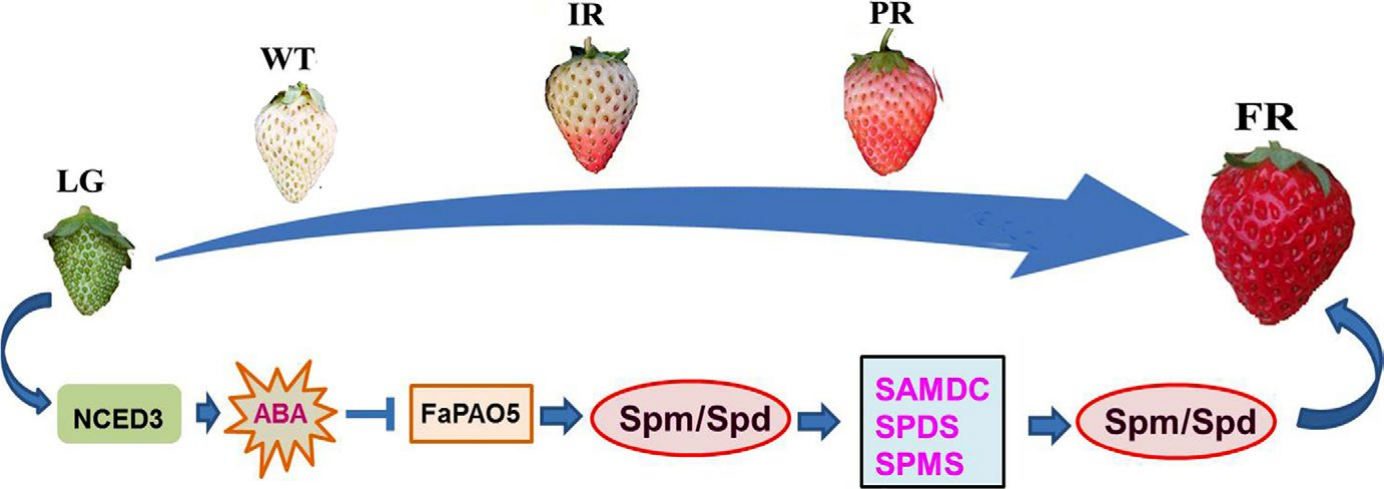
Model for FaPAO5‐mediated regulation of strawberry fruit ripening via ABA. Strawberry fruit ripening from large green (LG) to white (Wt), initially red (IR), partially red (PR), and fully red (FR) fruit stages. NCED3 promotes ABA accumulation, which inhibits *FaPAO5* expression and Spm/Spd accumulation, thereby promoting *SAMDC*, *SPDS*, and *SPMS* expression, Spm/Spd accumulation, and fruit ripening. The graphic symbols (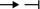) indicate induced and inhibited, respectively

The results of previous investigations and the current study indicate that: (a) ABA is important for strawberry fruit ripening (Jia et al., 2011) and *NCED3* was screened based on the RNA‐seq analysis of RNAi fruits (Table S2); (b) ABA inhibits *FaPAO*5 expression while Spd and Spm promote *FaPAO*5 expression (Figure 2); (c) inhibiting FaPAO5 activity with guazatine or FaPAO5 expression by RNAi enhances ripening (Figures 2 and 3); (d) Manipulation of *FaPAO5* expression affects the expression of many genes (Figure 5 and Table S1), with downregulated *FaPAO5* expression promoting *SAMDC*, *SPDS*, and *SPMS* expression (Figure 5); and (e) FaPAO5 specifically and strongly binds both Spm and Spd (Figures 6 and 7). We developed a model for the PA regulation of strawberry fruit ripening as part of a signaling event. With the onset of ripening, NCED3 promotes the rapid accumulation of ABA, which may inhibit *FaPAO5* expression, leading to Spm/Spd accumulation. The increased Spm/Spd may trigger *SAMDC*, *SPDS*, and *SPMS* expression, further promoting Spm/Spd accumulation, which ultimately accelerates strawberry fruit ripening (Figure 8). Future investigations should aim to uncover the molecular mechanism underlying the ABA‐mediated inhibition of *FaPAO5* expression.

## Conflict of Interest

The authors declare no conflict of interest associated with the work described in this manuscript.

## Supporting information

Figure S1‐S8

## References

[pld3217-bib-0001] Agudelo‐Romero, P. , Ali, K. , Choi, Y. H. , Sousa, L. , Verpoorte, R. , Tiburcio, A. F. , & Fortes, A. M. (2014). Perturbation of polyamine catabolism affects grape ripening of *Vitis vinifera* cv. Trincadeira. Plant Physiology and Biochemistry, 74, 141–155. 10.1016/j.plaphy.2013.11.002 24296250

[pld3217-bib-0002] Alexander, L. , & Grierson, D. (2002). Ethylene biosynthesis and action in tomato: A model for climacteric fruit ripening. Journal of Experimental Botany, 53, 2039–2055. 10.1093/jxb/erf072 12324528

[pld3217-bib-0003] Bregoli, A. M. , Scaramagli, S. , Costa, G. , Sabatini, E. , Ziosi, V. , Biondi, S. , & Torrigiani, P. (2002). Peach (*Prunus persica*) fruit ripening: Aminoethoxyvinylglycine (AVG) and exogenous polyamines affect ethylene emission and flesh firmness. Physiologia Plantarum, 114, 472–481. 10.1034/j.1399-3054.2002.1140317.x 12060270

[pld3217-bib-0004] Chen, B. X. , Li, W. Y. , Gao, Y. T. , Chen, Z. J. , Zhang, W. N. , Liu, Q. J. … Liu, J. (2016). Involvement of polyamine oxidase‐produced hydrogen peroxide during coleorhiza‐limited germination of rice seeds. Frontiers in Plant Science, 7, 1219 10.3389/fpls.2016.01219 27570530 PMC4981591

[pld3217-bib-0005] Cohen, E. , Arad, S. M. , Heimer, Y. M. , & Mizrahi, Y. (1982). Participation of ornithine decarboxylase in early stages of tomato fruit development. Plant Physiology, 70, 540–543. 10.1104/pp.70.2.540 16662530 PMC1067184

[pld3217-bib-0006] Cona, A. , Rea, G. , Angelini, R. , Federico, R. , & Tavladoraki, P. (2006). Functions of amine oxidases in plant development and defense. Trends in Plant Science, 11, 80–88. 16406305 10.1016/j.tplants.2005.12.009

[pld3217-bib-0007] De Dios, P. , Matilla, A. J. , & Gallardo, M. (2006). Flower fertilization and fruit development prompt changes in free polyamines and ethylene in damson plum (*Prunus insititia* L.). Journal of Plant Physiology, 163, 86–97. 10.1016/j.jplph.2005.03.007 16360807

[pld3217-bib-0008] Dibble, A. R. , Davies, P. J. , & Mutschler, M. A. (1988). Polyamine content of long‐keeping alcobaca tomato fruit. Plant Physiology, 86, 338–340. 10.1104/pp.86.2.338 16665907 PMC1054483

[pld3217-bib-0009] Ebeed, H. T. , Hassan, N. M. , & Aljarani, A. M. (2017). Exogenous applications of polyamines modulate drought responses in wheat through osmolytes accumulation, increasing free polyamine levels and regulation of polyamine biosynthetic genes. Plant Physiology and Biochemistry, 118, 438–448. 10.1016/j.plaphy.2017.07.014 28743037

[pld3217-bib-0010] Fortes, A. M. , & Agudelo‐Romero, P. (2018). Polyamine metabolism in climacteric and non‐climacteric fruit ripening. Methods in Molecular Biology, 1694, 433–447. 29080186 10.1007/978-1-4939-7398-9_36

[pld3217-bib-0011] Fortes, A. M. , Teixeira, R. T. , & Agudelo‐Romero, P. (2015). Complex interplay of hormonal signals during grape berry ripening. Molecules, 20, 9326–9343. 10.3390/molecules20059326 26007186 PMC6272489

[pld3217-bib-0012] Gapper, N. E. , McQuinn, R. P. , & Giovannoni, J. J. (2013). Molecular and genetic regulation of fruit ripening. Plant Molecular Biology, 82, 575–591. 10.1007/s11103-013-0050-3 23585213

[pld3217-bib-0013] Guo, J. , Wang, S. , Yu, X. , Dong, R. , Li, Y. , Mei, X. , & Shen, Y. (2018). Polyamines regulate strawberry fruit ripening by abscisic acid, auxin, and ethylene. Plant Physiology, 177, 339–351. 10.1104/pp.18.00245 29523717 PMC5933135

[pld3217-bib-0014] Gupta, A. , Pal, R. K. , & Rajam, M. V. (2013). Delayed ripening and improved fruit processing quality in tomato by RNAi‐mediated silencing of three homologs of 1‐aminopropane‐1‐carboxylate synthase gene. Journal of Plant Physiology, 170, 987–995. 10.1016/j.jplph.2013.02.003 23507024

[pld3217-bib-0015] Handa, A. K. , Fatima, T. , & Mattoo, A. K. (2018). Polyamines: Bio‐molecules with diverse functions in plant and human health and disease. Frontiers in Chemistry, 6, 10 10.3389/fchem.2018.00010 29468148 PMC5807879

[pld3217-bib-0016] Handa, A. K. , & Mattoo, A. K. (2010). Differential and functional interactions emphasize the multiple roles of polyamines in plants. Plant Physiology and Biochemistry, 48, 540–546. 10.1016/j.plaphy.2010.02.009 20227284

[pld3217-bib-0017] Hao, Y. , Huang, B. , Jia, D. , Mann, T. , Jiang, X. , Qiu, Y. , … Liu, T. (2018). Identification of seven polyamine oxidase genes in tomato (*Solanum lycopersicum*) and their expression profiles under physiological and various stress conditions. Journal of Plant Physiology, 228, 1–11. 29793152 10.1016/j.jplph.2018.05.004

[pld3217-bib-0018] Huang, Y. , Xu, P. H. , Hou, B. Z. , & Shen, Y. Y. (2019). Strawberry tonoplast transporter, FaVPT1, controls phosphate accumulation and fruit quality. Plant, Cell and Environment, 42, 2715–2729. 10.1111/pce.13598 31151133

[pld3217-bib-0019] Jia, H. F. , Chai, Y. M. , Li, C. L. , Lu, D. , Luo, J. J. , Qin, L. , & Shen, Y. Y. (2011). Abscisic acid plays an important role in the regulation of strawberry fruit ripening. Plant Physiology, 157, 188–199. 10.1104/pp.111.177311 21734113 PMC3165869

[pld3217-bib-0020] Jia, H. F. , Lu, D. , Sun, J. H. , Li, C. L. , Xing, Y. , Qin, L. , & Shen, Y. Y. (2013). Type 2C protein phosphatase ABI1 is a negative regulator of strawberry fruit ripening. Journal of Experimental Botany, 64, 1677–1687. 10.1093/jxb/ert028 23404898 PMC3617833

[pld3217-bib-0021] Jia, M. , Ding, N. , Zhang, Q. , Xing, S. , Wei, L. , Zhao, Y. , … Jia, W. (2017). A FERONIA‐like receptor kinase regulates strawberry (*Fragaria* × *ananassa*) fruit ripening and quality formation. Frontiers in Plant Science, 8, 1099 10.3389/fpls.2017.01099 28702036 PMC5487432

[pld3217-bib-0022] Jiang, J. M. , Lin, Y. X. , Chen, Y. Y. , Deng, C. J. , Gong, H. W. , Xu, Q. Z. , … Chen, W. (2015). Proteomics approach reveals mechanism underlying susceptibility of loquat fruit to sunburn during color changing period. Food Chemistry, 176, 388–395. 10.1016/j.foodchem.2014.12.076 25624247

[pld3217-bib-0023] Kitashiba, H. , Hao, Y. J. , Honda, C. , & Moriguchi, T. (2005). Two types of spermine synthase gene: MdACL5 and MdSPMS are differentially involved in apple fruit development and cell growth. Gene, 361, 101–111. 10.1016/j.gene.2005.07.013 16182474

[pld3217-bib-0024] Koushesh, S. , Arzani, K. , & Barzegar, M. (2012). Postharvest polyamine application alleviates chilling injury and affects apricot storage ability. Journal of Agriculture and Food Chemistry, 60, 8947–8953. 10.1021/jf302088e 22867007

[pld3217-bib-0025] Kushad, M. M. , Yelenosky, G. , & Knight, R. (1988). Interrelationship of polyamine and ethylene biosynthesis during avocado fruit development and ripening. Plant Physiology, l87, 463–467. 10.1104/pp.87.2.463 PMC1054775 16666165

[pld3217-bib-0026] Lasanajak, Y. , Minocha, R. , Minocha, S. C. , Goyal, R. , Fatima, T. , Handa, A. K. , & Mattoo, A. K. (2014). Enhanced flux of substrates into polyamine biosynthesis but not ethylene in tomato fruit engineered with yeast S‐adenosylmethionine decarboxylase gene. Amino Acids, 46, 729–742. 10.1007/s00726-013-1624-8 24337930

[pld3217-bib-0027] Lester, G. E. (2000). Polyamines and their cellular anti‐senescence properties in honey dew muskmelon fruit. Plant Science, 160, 105–112. 10.1016/S0168-9452(00)00369-1 11164582

[pld3217-bib-0028] Li, C. L. , Jia, H. F. , Chai, Y. M. , & Shen, Y. Y. (2011). Abscisic acid perception and signaling transduction in strawberry: A model for non‐climacteric fruit ripening. Plant Signaling & Behavior, 6, 1950–1953. 10.4161/psb.6.12.18024 22095148 PMC3337185

[pld3217-bib-0029] Liu, M. , Pirrello, J. , Chervin, C. , Roustan, J. P. , & Bouzayen, M. (2015). Ethylene control of fruit ripening: Revisiting the complex network of transcriptional regulation. Plant Physiology, 169, 2380–2390. 26511917 10.1104/pp.15.01361 PMC4677914

[pld3217-bib-0030] Liu, T. , Huang, B. , Chen, L. , Xian, Z. , Song, S. , Chen, R. , & Hao, Y. (2018). Genome‐wide identification, phylogenetic analysis, and expression profiling of polyamine synthesis gene family members in tomato. Gene, 661, 1–10. 10.1016/j.gene.2018.03.084 29605609

[pld3217-bib-0031] Livak, K. J. , & Schmittgen, T. D. (2001). Analysis of relative gene expression data using real‐time quantitative PCR and the 2(‐Delta Delta C (T)) method. Methods, 25, 402–408. 11846609 10.1006/meth.2001.1262

[pld3217-bib-0032] Mattoo, A. K. , Chung, S. H. , Goyal, R. K. , Fatima, T. , Solomos, T. , Srivastava, A. , & Handa, A. K. (2007). Overaccumulation of higher polyamines in ripening transgenic tomato fruit revives metabolic memory, upregulates anabolism‐related genes, and positively impacts nutritional quality. Journal of AOAC International, 90, 1456–1464. 10.1093/jaoac/90.5.1456 17955994

[pld3217-bib-0033] Mattoo, A. K. , Sobolev, A. P. , Neelam, A. , Goyal, R. K. , Handa, A. K. , & Segre, A. L. (2006). Nuclear magnetic resonance spectroscopy‐based metabolite profiling of transgenic tomato fruit engineered to accumulate spermidine and spermine reveals enhanced anabolic and nitrogen‐carbon interactions. Plant Physiology, 142, 1759–1770. 10.1104/pp.106.084400 17041034 PMC1676058

[pld3217-bib-0034] Mehta, R. A. , Cassol, T. , Li, N. , Ali, N. , Handa, A. K. , & Mattoo, A. K. (2002). Engineered polyamine accumulation in tomato enhances phytonutrient content, juice quality, and vine life. Nature Biotechnology, 20, 613–618. 10.1038/nbt0602-613 12042867

[pld3217-bib-0035] Minocha, R. , Majumdar, R. , & Minocha, S. C. (2014). Polyamines and abiotic stress in plants: A complex relationship. Frontiers in Plant Science, 5, 1–17. 10.3389/fpls.2014.00175 PMC4017135 24847338

[pld3217-bib-0036] Moschou, P. N. , Sanmartin, M. , Andriopoulou, A. H. , Rojo, E. , Sanchez‐Serrano, J. J. , & Roubelakis‐Angelakis, K. A. (2008). Bridging the gap between plant and mammalian polyamine catabolism: A novel peroxisomal polyamine oxidase responsible for a full back‐conversion pathway in Arabidopsis. Plant Physiology, 147, 1845–1857. 18583528 10.1104/pp.108.123802 PMC2492618

[pld3217-bib-0037] Mutschler, M. A. (1984). Inheritance and linkage of the Alcobaca ripening mutant in tomato. American Society for Horticultural Science, 109, 500–503.

[pld3217-bib-0038] Pandey, R. , Gupta, A. , Chowdhary, A. , Pal, R. K. , & Rajam, M. V. (2015). Over‐expression of mouse ornithine decarboxylase gene under the control of fruit‐specific promoter enhances fruit quality in tomato. Plant Molecular Biology, 87, 249–260. 10.1007/s11103-014-0273-y 25537646

[pld3217-bib-0039] Parra, L. M. C. , & Gomez, J. M. C. (2011). Polyamine‐induced modulation of genes involved in ethylene biosynthesis and signalling pathways and nitric oxide production during olive mature fruit abscission. Journal of Experimental Botany, 62, 4447–4465. 10.1093/jxb/err124 21633085 PMC3170544

[pld3217-bib-0040] Rodriguez, S. C. , López, B. , & Chaves, A. R. (1999). Changes in polyamines and ethylene during the development and ripening of eggplant fruits *(Solanum melongena*). Journal of Agriculture and Food Chemistry, 47, 1431–1434. 10.1021/jf980997d 10563994

[pld3217-bib-0041] Romero, F. M. , Maiale, S. J. , Rossi, F. R. , Marina, M. , Ruíz, O. A. , & Gárriz, A. (2018). Polyamine metabolism responses to biotic and abiotic stress. Methods in Molecular Biology, 1694, 37–49. 29080153 10.1007/978-1-4939-7398-9_3

[pld3217-bib-0042] Rose, J. K. C. , & Shen, Y. Y. (2014) ABA metabolism and signaling in fleshy fruits In: Abscisic acid: Metabolism, transport and signaling. New York, NY: Springer, pp. 271–286.

[pld3217-bib-0043] Saftner, R. A. , & Baldi, B. G. (1990). Polyamine levels and tomato fruit development: Possible interaction with ethylene. Plant Physiology, 92, 547–550. 10.1104/pp.92.2.547 16667313 PMC1062329

[pld3217-bib-0044] Sharma, S. , Pareek, S. , Sagar, N. A. , Valero, D. , & Serrano, M. (2017). Modulatory effects of exogenously applied polyamines on postharvest physiology, antioxidant system and shelf Life of fruits: A review. International Journal of Molecular Sciences, 18(8), 1789 10.3390/ijms18081789 PMC5578177 28817100

[pld3217-bib-0045] Shiozaki, S. , Ogata, T. , & Horiuchi, S. (2000). Endogenous polyamines in the pericarp and seed of the grape berry during development and ripening. Scientia Horticulturae, 83, 33–41. 10.1016/S0304-4238(99)00064-3

[pld3217-bib-0046] Simpson, C. G. , Cullen, D. W. , Hackett, C. A. , Smith, K. , Hallett, P. D. , McNicol, J. , … Graham, J. (2017). Mapping and expression of genes associated with raspberry fruit ripening and softening. TAG. Theoretical and Applied Genetics, 130, 557–572. 10.1007/s00122-016-2835-7 27942774

[pld3217-bib-0047] Tassoni, A. , Watkins, C. B. , & Davies, P. J. (2006). Inhibition of the ethylene response by 1‐MCP in tomato suggests that polyamines are not involved in delaying ripening, but may moderate the rate of ripening or over‐ripening. Journal of Experimental Botany, 57, 3313–3325. 10.1093/jxb/erl092 16920766

[pld3217-bib-0048] Tavladoraki, P. , Rossi, M. N. , Saccuti, G. , Perez‐Amador, M. A. , Polticelli, F. , Angelini, R. , & Federico, R. (2006). Heterologous expression and biochemical characterization of a polyamine oxidase from arabidopsis involved in polyamine back conversion. Plant Physiology, 141, 1519–1532. 16778015 10.1104/pp.106.080911 PMC1533960

[pld3217-bib-0049] Teh, H. F. , Neoh, B. K. , Wong, Y. C. , Kwong, Q. B. , Ooi, T. E. , Ng, T. L. , … Appleton, D. R. (2014). Hormones, polyamines, and cell wall metabolism during oil palm fruit mesocarp development and ripening. Journal of Agriculture and Food Chemistry, 62, 8143–8152. 10.1021/jf500975h 25032485

[pld3217-bib-0050] Torrigiani, P. , Bressanin, D. , Ruiz, K. B. , Tadiello, A. , Trainotti, L. , Bonghi, C. , … Costa, G. (2012). Spermidine application to young developing peach fruits leads to a slowing down of ripening by impairing ripening‐related ethylene and auxin metabolism and signaling. Physiologia Plantarum, 146, 86–98. 10.1111/j.1399-3054.2012.01612.x 22409726

[pld3217-bib-0051] Tsaniklidis, G. , Kotsiras, A. , Tsafouros, A. , Roussos, P. A. , Aivalakis, G. , Katinakis, P. , & Delis, C. (2016). Spatial and temporal distribution of genes involved in polyamine metabolism during tomato fruit development. Plant Physiology and Biochemistry, 100, 27–36. 10.1016/j.plaphy.2016.01.001 26773542

[pld3217-bib-0052] Wang, Q. H. , Zhao, C. , Zhang, M. , Li, Y. Z. , Shen, Y. Y. , & Guo, J. X. (2017). Transcriptome analysis around the onset of strawberry fruit ripening uncovers an important role of oxidative phosphorylation in ripening. Scientific Reports, 7, 41477 10.1038/srep41477 28195221 PMC5307319

[pld3217-bib-0053] Wang, S. , Song, M. , Guo, J. , Huang, Y. , Zhang, F. , Xu, C. , … Zhang, L. (2018). The potassium channel FaTPK1 plays a critical role in fruit quality formation in strawberry (*Fragaria* × *ananassa*). Plant Biotechnology Journal, 16, 737–748. 28851008 10.1111/pbi.12824 PMC5814577

[pld3217-bib-0054] Wang, W. , & Liu, J. H. (2016). CsPAO4 of *Citrus sinensis* functions in polyamine terminal catabolism and inhibits plant growth under salt stress. Scientific Reports, 6, 31384 10.1038/srep31384 27535697 PMC4989168

[pld3217-bib-0055] Wild, R. , Gerasimaite, R. , Jung, J.‐Y. , Truffault, V. , Pavlovic, I. , Schmidt, A. , … Mayer, A. (2016). Control of eukaryotic phosphate homeostasis by inositol polyphosphate sensor domains. Science, 352, 986–990. 10.1126/science.aad9858 27080106

[pld3217-bib-0056] Winer, L. , Vinkler, C. , & Apelbaum, A. (1984). Partial purification and characterization of arginine decarboxylase from avocado fruit, a thermostable enzyme. Plant Physiology, 76, 233–237. 10.1104/pp.76.1.233 16663805 PMC1064262

[pld3217-bib-0057] Xue, B. , Zhang, A. , & Jiang, M. (2009). Involvement of polyamine oxidase in abscisic acidinduced cytosolic antioxidant defense in leaves of maize. Journal of Integrative Plant Biology, 51, 225–234. 19261065 10.1111/j.1744-7909.2008.00766.x

[pld3217-bib-0058] Zahedi, S. M. , Hosseini, M. S. , Karimi, M. , & Ebrahimzadeh, A. (2019). Effects of postharvest polyamine application and edible coating on maintaining quality of mango (*Mangifera indica* L.) cv. Langra during cold storage. Food Sciences and Nutrition, 7, 433–441. 10.1002/fsn3.802 PMC6393044 30847120

[pld3217-bib-0059] Zarza, X. , Atanasov, K. E. , Marco, F. , Arbona, V. , Carrasco, P. , Kopka, J. , … Alcázar, R. (2017). Polyamine oxidase 5 loss‐of‐function mutations in Arabidopsis thaliana trigger metabolic and transcriptional reprogramming and promote salt stress tolerance. Plant, Cell and Environment, 40, 527–542. 10.1111/pce.12714 26791972

[pld3217-bib-0060] Zhang, Z. , Honda, C. , Kita, M. , Hu, C. , Nakayama, M. , & Moriguchi, T. (2003). Structure and expression of spermidine synthase genes in apple: Two cDNAs are spatially and developmentally regulated through alternative splicing. Molecular Genetics and Genomics, 268, 799–807. 12655406 10.1007/s00438-002-0802-2

