# Supplementary material for: FaPAO5 regulates Spm/Spd levels as a signaling during strawberry fruit ripening
Source: Plant Direct. 2020 Apr 29;4(5):e00217. doi: 10.1002/pld3.217 (PMC7189608; doi:10.1002/pld3.217)
Supplement: Supplementary file 1 — Figure S1‐S8 [file PLD3-4-e00217-s001.docx]

**Supplemental files**

**Supplemental Materials and Methods**

**Expression and Purification of FaPAO5 Recombinant Proteins**

Expression and purification of FaPAO5 were done in an *E.coli* expression system. The coding sequence of *FaPAO5* was amplified by PCR (forward,5’-AAGGAAAAAAGGCGGCCGCATGGTGGCCAAGAAACCAAGAATTG-3’, *Not*I site underlined; reverse, 5’-ACGCGTCGACAACCCCAACATGGTGGTTG-3’, *Sal*I site underlined) and cloned into the expression vector pMAL-C5X-MBP-His in frame with the N-terminal MBP fusion tag, which was transformed into *E.coli* to enable the selection of transformants on LB plates containing 100 μg/mL ampicillin. Ten transformants were selected to confirm the correct fusion frame by sequencing. The purified recombinant plasmids were transformed into *E.coli* BL21 to select the ampicillin-resistant *E.coli* transformants. The FaPAO5-MBP fusion protein was expressed at 16 °C in LB broth with 0.5 mM IPTG for 6 h. The purification of the FaPAO5-MBP fusion protein was carried using Amylose Resin (NEB, Beijing), and immunoblotting was performed with an antibody of the C-terminal His tag using a one-step western kit HRP (antiMouse; Kangwei Company) following the manufacture’s protocols. The eluted fusion protein was stored at -80 °C until use.

**Effects of pH and temperature on FaPAO5 activity with polyamine by H_2_O_2_ production**

The catalytic activities of recombinant FaPAO5 protein for the oxidation of Put, Spd and Spm were determined as described by Wang et al. (2016). In order to determine the optimum pH, 100 mM MES buffer for the pH 4.0-5.0 range and 100 mM phosphate buffer for pH 5.5-9.0 range were used. To investigate the optimum temperature, the temperature from 20 °C to 45 °C was determined with the optimum pH have been confirmed above. In a typical experiment, about 5μg of protein was added to a buffered solution containing the substrate (500 μM), 4-aminoantipyrine (100 μM), 3, 5-dichloro-2-hydroxybenzesulfonic acid (1 mM), and horseradish peroxidase (10 U/ml), and the increase in absorbance at 515 nm was monitored.

**HPLC analysis of FaPAO5 activity**

To determine the reaction products of PAs oxidation (Wang et al, 2016), 10 μg of purified FaPAO5 protein was incubated with 150 mM Spd in 100mM phosphate buffer (pH 7.5) at 38 °C or with 150 mM Spm in 100 mM phosphate buffer (pH 6.5) at 36 °C for different time points. The reaction products were extracted by Precooled perchlorate (volume fraction 5%) ,The derivation and benzoylation of the extracted PAs and Dap were performed . The contents were examined by HPLC using the C18-column (Zorbax Eclipse XDB-C18, 4.6 × 250 mm, 5 μm; Agilent) at 30 °C. The mobile phase was methanol:water (v:v, 64:36) for 20 min at a flow rate of 0.7 mL per min.Effluent absorbance was monitored at 230 nm. The injection volume was 10 mL. The experiment was repeated three times.

**Supplemental Tables**

**Supplemental Table S1:** Differential expression unigenes and top6 pathways in the comparison of RNAi to control fruits


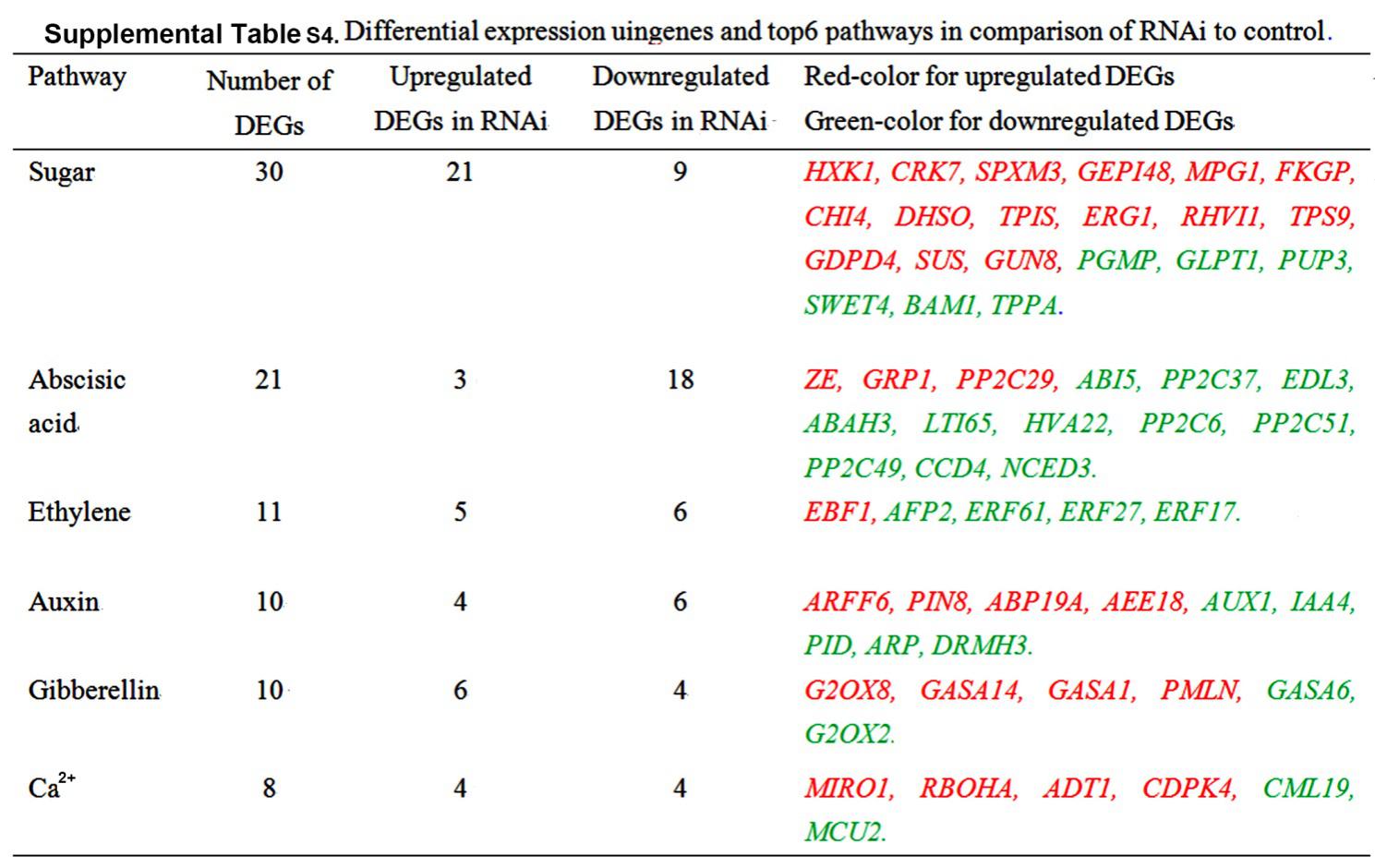


**Supplemental Table S2:** The screened unigenes were blasted and annotated by the transcriptome data around the onset of strawberry fruit ripening including LG, Wt, IR and PR (Bioproject accession in GenBank: PRJNA438551).

| Uingene name | Relative expression levels of uingenes during ripening | | | | Number in NCBI |
| --- | --- | --- | --- | --- | --- |
|  | LG | WT | IR | PR |  |
| HXK1: hexokinase1gene; Kiryakova et al., 2016 | 4.25 | 2.09 | 2.9 | 2.39 | XP_004302852.1 |
| CRK7: cysteine rich kinase 7 gene; Acharya et al., 2010 |  |  |  |  | XP_024167065.1 |
| SPXM3: SPX domain containing membrane protein gene; Secco et al., 2012 | 0.58 | 0.09 | 0.2 | 0.02 | XP_004298733.1 |
| GEPI48: UDP glucose 4 epimerase gene; Dörmann et al., 2010 | 5.83 | 5.2 | 6.99 | 9.2 | XP_004298907.1 |
| *MPG1*: mannose 1 phosphate guanylyltransferase 1 gene; Kumar et al., 2012 | 29.98 | 23.1 | 22.49 | 21.65 | XP_004307574.1 |
| *FKGP*: bifunctional fucokinase/fucose pyrophosphorylase gene; Kotake et al., 2008 | 8.64 | 8.34 | 7.69 | 6.57 | XP_011468916.1 |
| *CHI4*: chitinase 4 gene; Pappinen et al., 2002 | 3.89 | 3.8 | 2.36 | 4.49 | XP_004309834.1 |
| *DHSO*: sorbitol dehydrogenase gene; Jose et al., 2010 |  |  |  |  | XP_004308124.1 |
| *TPIS*: triosephosphate isomerase gene; | 4.09 | 1.94 | 2.27 | 1.96 | XP_004307511.1 |
| *ERG1*: squalene monooxygenase gene; Tsai et al., 2004 | 3.55 | 0.83 | 1.33 | 0.97 | XP_004289714.2 |
| *ZE*: zeaxanthin epoxidase gene; Burbidge et al., 1997 |  |  |  |  | XP_004288322.1 |
| *RHVI1*: beta-fructofuranosidase gene; Farci et al., 2016 |  |  |  |  | XP_004307805.1 |
| *TPS9*: protein nuclear fusion defective 2 gene; Ze et al., 2017 | 0.25 | 0.13 | 0.38 | 0.72 | XP_004298929.1 |
| *GDPD4*: Glycerophosphodiester phosphodiesterase gene; Sousa et al., 2013 |  |  |  |  | XP_011457810.1 |
| *SUS*: sucrose synthase gene; Bieniawska et al., 2010 | 2.88 | 1.69 | 0.81 | 0.35 | XP_004287669.1 |
| *GUN8*: endo 1,4 beta-glucanase gene; | 18.42 | 92.48 | 180.67 | 326.04 | AAQ15175.1 |
| *PGMP*: phosphoglucomutase chloroplastic gene; Barojafernández et al., 2001 | 15.19 | 10.37 | 8.7 | 5.86 | XP_004308130.1 |
| *GLPT1*: putative glycerol 3 phosphate transporter 1 gene; Huang et al., 2003 | 0.89 | 3.6 | 6.57 | 8.19 | XP_011470080.1 |
| *PUP3*: purine permease 3 gene; Zhang et al., 2015 | 0.57 | 0.12 | 0 | 0.11 | XP_011464628.1 |
| *SWET4*: bidirectional sugar transporter gene; Fang et al., 2017 | 10.25 | 4.38 | 2.97 | 2.8 | XP_004300494.1 |
| *BAM1*: beta-amylase 1 gene; Hord et al., 2006 | 5.66 | 5.88 | 4.25 | 3.53 | XP_004296549.1 |
| *TPPA*: trehalose-phosphate phosphatase A gene; Vogel et al., 2010 | 30.51 | 22.89 | 24.61 | 14.27 | XP_004307816.1 |
| *GRP1*: glycine-rich RNA-binding protein gene; Nishiyama et al., 1997 | 3.66 | 6.38 | 4.08 | 3.7 | XP_004289673.1 |
| *PP2C29*: protein phosphatase 2C 29 gene; Merlot et al., 2010 | 0.47 | 0.74 | 0.7 | 1 | XP_011465169.1 |
| *ABI5*: abscisic acid insensitive 5 gene; Carles et al., 2002 | 6.53 | 6.44 | 5.01 | 4.74 | XP_004291101.1 |
| *PP2C37*: protein phosphatase 2C 37 gene; Merlot et al., 2010 | 19.03 | 31.51 | 26.15 | 24.35 | XP_004307470.1 |
| *EDL3*: Eid1 F-box protein 3 gene; Koops et al., 2011 | 0.75 | 2.87 | 2.76 | 1.19 | XP_004292601.1 |
| *ABAH3*: abscisic acid 8'-hydroxylase 4 gene; Okamoto et al., 2009 | 0.62 | 0.38 | 0.26 | 0.25 | XP_004291107.1 |
| *LTI65*: low temperature-induced 65 kDa protein gene; Nordin et al., 1993 |  |  |  |  | XP_011470368.1 |
| *HVA22*: HVA22 protein gene; Shen et al., 2001 | 0.27 | 0.28 | 0.26 | 0.22 | XP_004297524.1 |
| *PP2C6*: protein phosphatase 2C 6 gene; Merlot et al., 2010 | 2.26 | 1.97 | 1.77 | 1.61 | XP_004287470.1 |
| *PP2C51*: protein phosphatase 2C 51 gene; Merlot et al., 2010 |  |  |  |  | XP_004307973.1 |
| *PP2C49*: protein phosphatase 2C 49 gene; Merlot et al., 2010 | 1.53 | 1.69 | 2.41 | 3.26 | XP_004289425.1 |
| *CCD4*: carotenoid cleavage dioxygenase 4 gene; Adami et al., 2013 | 16.76 | 13.56 | 8.55 | 3.03 | XP_004297644.1 |
| *NCED3*: 9-cis-epoxycarotenoid dioxygenase gene; Niu et al., 2015 | 11.07 | 71.35 | 109.27 | 76.29 | XP_004300667.1 |
| *EBF1*: EIN3-binding F-box protein 1 gene; Gagne et al., 2004 | 8.39 | 23.1 | 10.39 | 9.1 | XP_004287307.1 |
| *AFP2*: ninja-family gene; | 4.21 | 3.24 | 3.38 | 2.53 | XP_004297228.1 |
| *ERF61*: ethylene responsive transcription factor ERF061 gene; Thirugnanas et al., 2015 | 0.59 | 4.36 | 10.69 | 29.55 | XP_004298895.1 |
| *ERF27*: ethylene responsive transcription factor ERF027 gene; Thirugnanas et al., 2015 |  |  |  |  | XP_004298771.1 |
| ERF17: ethylene responsive transcription factor ERF017 gene; Thirugnanas et al., 2015 | 0.6 | 0.14 | 0 | 0 | XP_004287662.1 |
| *ARF6*: auxin response factor 6 gene; Tabata et al., 2010 | 18.61 | 18.53 | 19.48 | 16.65 | XP_004293501.1 |
| *PIN8*: putative auxin efflux carrier component 8 gene; Chawla et al., 2004 | 1.43 | 1.04 | 0.58 | 0.34 | XP_004302153.1 |
| *ABP19A*: auxin-binding protein ABP19a gene; Effendi et al., 2013 | 0.27 | 0.18 | 0.13 | 0.67 | XP_004288274.1 |
| *AEE18*: acyl-activating enzyme 18 gene; Shockey et al., 2003 | 4.79 | 3.3 | 3.12 | 3.75 | XP_004294995.1 |
| *AUX1*: AUX1 gene;Timpte et al., 2010 | 0.38 | 0.68 | 1.25 | 1.71 | XP_004290835.1 |
| *IAA4*: auxin-responsive protein IAA4 gene; Wong et al., 2010 | 32.59 | 5.16 | 1.53 | 0.64 | XP_024186399.1 |
| *PID*: protein kinase pinoid gene; Benjamins et al., 2001 | 2.54 | 8.96 | 11.18 | 12.36 | XP_004304710.1 |
| *ARP*: Auxin-repressed 12.5 kDa protein gene; Zhao et al., 2014 | 109.27 | 36.19 | 3.93 | 4.11 | Q05349.1 |
| *DRMH3*: F-box/kelch-repeat gene; Curtis et ao.,2013 |  |  |  |  | XP_004302272.1 |
| *G2OX8*: gibberellin 2-beta-dioxygenase 8 gene; Schomburg et al., 1994 | 0.35 | 0.32 | 0.11 | 0.11 | XP_004297767.1 |
| *GASA14*:gibberellin-regulated protein 14 gene; Aubert ET AL., 1998 |  |  |  |  | XP_011463517.1 |
| GASA1:gibberellin-regulated protein 1 gene; Aubert ET AL., 1998 | 1.65 | 4.18 | 6.43 | 7.19 | XP_004291363.1 |
| *PMLN*: peamaclein gene; |  |  |  |  | XP_004290947.1 |
| *GASA6*: gibberellin-regulated protein 1 gene; Aubert ET AL., 1998 | 190.04 | 49.55 | 15.66 | 21.19 | XP_004304306.1 |
| *G2OX2*: gibberellin 2-beta-dioxygenase 2 gene; Schomburg et al., 1994 | 0 | 1.12 | 0 | 0.4 | XP_004308939.2 |
| *MIRO1*: mitochondrial Rho GTPase 1 gene; Yamaoka et al., 2011 | 21.22 | 14.91 | 14.49 | 13.92 | XP_004302432.1 |
| *RBOHA*: respiratory burst oxidase homolog protein A gene; Wang et al; 2015 | 0.02 | 0.04 | 0.83 | 1.21 | XP_011464313.1 |
| *ADT1*: transmembrane transporter activity gene; | 198.89 | 217.66 | 215.29 | 207.36 | XP_023729770.1 |
| *CDPK4*: calcium-dependent protein kinase 26 gene; Yang et al., 2012 | 24.92 | 22.82 | 23.19 | 27.08 | XP_004308122.1 |
| *CML19*: putative calcium-binding gene; Lokdarshi et al., 2016 | 0.02 | 0.31 | 0 | 0.57 | XP_011464987.1 |
| *MCU2*: calcium uniporter 2 gene; De et al., 2011 | 1.52 | 0.54 | 0.53 | 0.62 | XP_004300595.2 |

**Supplemental Table S3**: Primers used to amplify *FaPAO* and vector construction.

| **Gene** | **Forward primer (5’-3’)** | **Reverse primer (5’-3’)** | **GenBank**  **accession number** |
| --- | --- | --- | --- |
| *FaPAO5* | ATGGTGGCCAAGAAACCAAGAATTG | CTAAACCCCAACATGGTGGTTG | XM_004307176 |
| *FaPAO5*-gateway-OE | GGGGACAAGTTTGTACAAAAAAGCAGGCTTCATGGTGGCCAAGAAACCAAGAATTG | GGGGACCACTTTGTACAAGAAAGCTGGGTCAACCCCAACATGGTGGTTG | XM_004307176 |
| *FaPAO5*-gateway-RNAi | GGGGACAAGTTTGTACAAAAAAGCAGGCTTCACTCAAAGAT GAAGAGAT | GGGGACCACTTTGTACAAGAAAGCTGGGTCAACCCCAACATGGTGGTTG | XM_004307176 |
| *FaPAO5-*Super1300 | GGTCGACATTTAAATACTAGTATGGTGGCCAAGAAACCAAGAATTG | GCCCTTGCTCACCATGGTACCAACCCCAACATGGTGGTTG | XM_004307176 |
| *FaPAO5*-C5X-MBP-His | AAGGAAAAAAGGCGGCCGCATGGTGGCCAAGAAACCAAGAATTG | ACGCGTCGACAACCCCAACATGGTGGTTG | XM_004307176 |
| *FaPAO1* | ATGGACTCTCCTCATCGCTCC | ATATAACTTGGGTTGTTTAGAGCC | XM_011471201 |
| *FaPAO1*-gateway-RNAi | GGGGACAAGTTTGTACAAAAAAGCAGGCTTCTACCTCACTGGGATCGACAC | GGGGACCACTTTGTACAAGAAAGCTGGGTCCTAATATAACTTGGGTTGTTTAGAGCCAA | XM_011471201 |
| *FaPAO1-*Super1300 | GCAAATCCTTGAATGGAGGAGGAGGAGGAGGTACCATGGACTCTCCTCATCGCTCC | GTTTGAACGATCGGGGAAATTCGAGCTCATATAACTTGGGTTGTTTAGAGCC | XM_011471201 |
| *FaPAO2* | ATGGCTTCCGGTAATAGCAAC | CAGGCGGGAGATCTGAATTGG | XM_004303856 |
| *FaPAO2*-gateway-RNAi | GGGGACAAGTTTGTACAAAAAAGCAGGCTTCATGGCTTCCGGTAATAGCAAC | GGGGACCACTTTGTACAAGAAAGCTGGGTCAACCTGAAATGAAGCATCGTGAAG | XM_004303856 |
| *FaPAO2-*Super1300 | AGAGCAAATCCTTGAATGGAGGAGGAGGAGGAGGTACCATGGCTTCCGGTAATAGCAAC | CAAATGTTTGAACGATCGGGGAAATTCGAGCTC CAGGCGGGAGATCTGAATTGG | XM_004303856 |
| *FaPAO4* | ATGGACCCCAAAGATCCGTTC | AATCCTTGAGATTTGTAGTGGAACTAT | XM_004288527 |
| *FaPAO4*-gateway-RNAi | GGGGACAAGTTTGTACAAAAAAGCAGGCTTCGGTAATCTTTTCTTTGGAGGGGAAG | GGGGACCACTTTGTACAAGAAAGCTGGGTCTCAAATCCTTGAGATTTGTAGTGGAACTAT | XM_004288527 |
| *FaPAO4-*Super1300 | AGCAAATCCTTGAATGGAGGAGGAGGAGGAGGTACCATGGACCCCAAAGATCCGTTC | TGTTTGAACGATCGGGGAAATTCGAGCTCAATCCTTGAGATTTGTAGTGGAAC | XM_004288527 |

**Supplemental Table S4**: Real-time PCR primers for polyamines.

| **Gene** | **Forward primer (5’-3’)** | **Reverse primer (5’-3’)** | **GenBank accession number** |
| --- | --- | --- | --- |
| *FaPAO5* | GTGCTACTTCAACTTCTTCA | CTAAACCCCAACATGGTGGT | XM_004307176 |
| *FaADC* | TATCGCAACCTTTCGGCT | TACCCAGAGACCCTTCCTT | XM_004306397 |
| *FaODC* | CAGAGACTCCTTTCACGCTTG | ATGCCGTCGTCACTGTTATTG | XM_011464632 |
| *FaSAMDC* | AGGAAGAAGGCATCTGTGTT | AGTTCATAGAGTAGCCGCAAG | XM_011464655 |
| *FaSPDS* | GTGAAGAGGGCGAGGGAG | CCAGGAATAACACCAGAGATACA | XM_004297595 |
| *FaSPMS* | ATGGGGGAAGACGCAG | TTCAAGCAACAAGAAGGGAT | XM_004307924 |

**Supplemental Table S5：**Real-time PCR primers for ripening-related genes.

| **Gene** | **Full Name** | **Forward primer (5’-3’)** | **Reverse primer (5’-3’)** | **GenBank accession number** |
| --- | --- | --- | --- | --- |
| *FaC4H* | cinnamate-4-hydroxylase | ACGCTCAACAGAAAGGAGAGAT | TTCGGGGTGGTTCACAA | DQ898278 |
| *Fa4CL1* | 4-coumarate:CoA ligase1 | CGTAGACCCTGAAACTGGTG | GTGTAGCCATCCTTCCTTGTC | XM_004303164 |
| *FaCHS* | chalcone synthase | CATACCCCGACTACTACTTTCGT | CGCACATACTGGGATTCTCTT | AY997297 |
| *FaCHI* | chalcone isomerase | AGCGAAAGCCATTGAAAAGT | CATTTGGTGATTGTGTGAAGAG | XM_004307403 |
| *FaF3H* | flavanone 3-hydroxylase | CTTTCGTGGTGAATCTTGGAG | TCGCTATGGACAACCTGCT | AY679608 |
| *FaDFR* | dihydroflavonol 4-reductase | ACCCTGAGAACGAAGTGATAAAG | TAAACACCACCCTCCGAACT | AY695813 |
| *FaUFGT* | flavonoid 3-O-glucosyltransferase | TAGAGGATGTGTGGAAGATTGGT | CTGTTGTGCGAGTTGTTTTAGTG | KP165417 |
| *FaANS* | anthocyanidin synthase | CTTGGCTTGGGATTAGAAGAAG | TGAGGGCATTTTGGGTAGTAGT | XM_024317695 |
| *FaPE* | pentaerythritol | GGTTTCTACTGGTGCTGGTTTT | CTCGGACTGTATCGTGTTGC | AY324809 |
| *FaPL* | pectate lyase | TCAACTCGTCAATGGCAGAC | GAATGCTCGTATCAACCAGAGA | DQ076239 |
| *FaPG* | polygalacturonase1 | GCAAGTAGAGTCGCACAGTTTT | TCAGTATTAGGCTTCCCACCA | AF380299 |
| *FaCEL* | carboxyl ester lipase | GCTCTGTTTTGCCTGGACTT | GCGTGGCTTAGATAGTTGGAAT | AF357596 |
| *FaXYL1* | alpha-xylosidase1 | ATGGAAAGCCTACTTGTGCTG | CTGGTGTAATGTTGTTGGTCGT | AY486104 |
| *FaEXP2* | expansin2 | GTATCGTCCCCGTCTCATTC | AGTAGGAGTGCCCGTTGATT | AF159563 |
| *FaSUT1* | sucrose transporter1 | TTCAAGCGACAGAAATACCC | ACCCAATCCAGTTTAGACCAG | JX013937 |
| *FaSS* | sucrose synthase | TTATCCCTCGCATTCTTATT | CAATTCCCTTCTCGGTTCTA | AB275666 |
| *FaNCED1* | 9-cis-epoxycarotenoid dioxygenase1 | ACGACTTCGCCATTACCG | AGCATCGCTCGCATTCT | HQ290318 |
| *FaABI37* | protein phosphatase 2C37 | GATTCGACGATCCCATCCGA | GGGTCAAGTTGGTGCCTTCA | XM_004307422 |
| *FaSnRK2.6* | SNF1-related protein kinase2.6 | GCTACACTCGCAACCAAAATC | ACCCCACAAGACCAGACATC | KJ748362 |
| *FaACTIN* | actin protein | TGCATATATCAAGCAACTTTACACTGA | ATAGCTGAGATGGATCTTCCTGT | XM_011470684 |


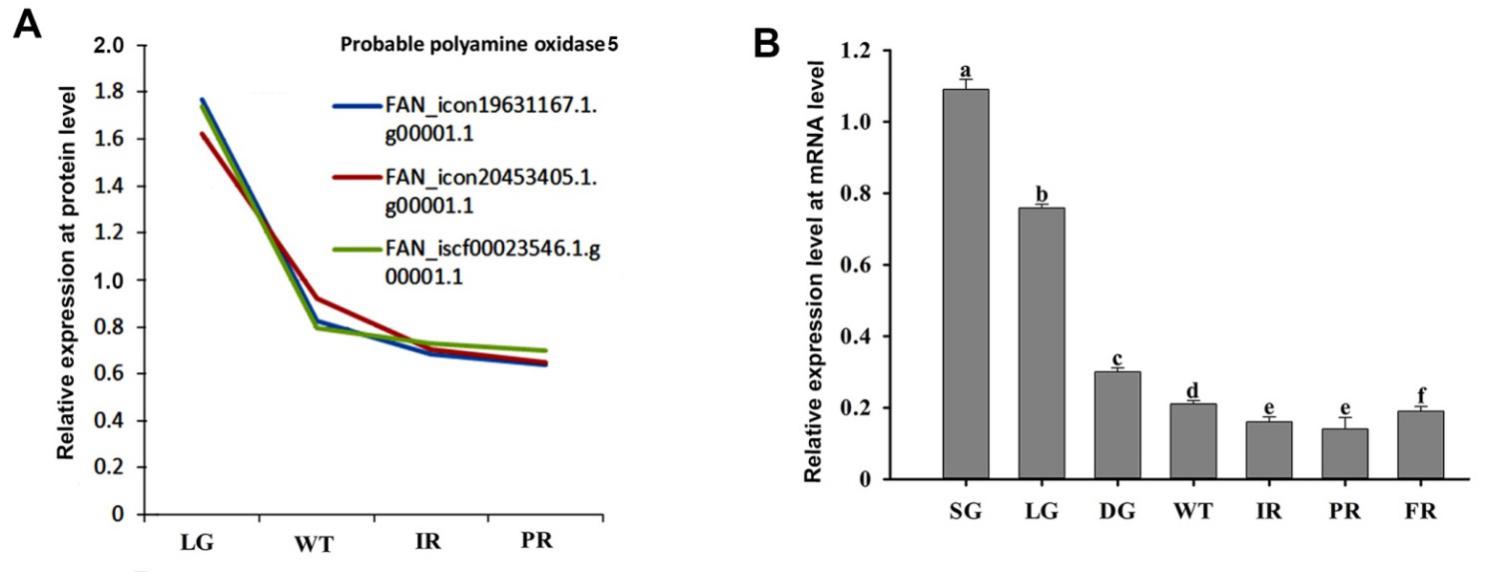


**Supplemental Figure S1**. FaPAO5 expression patter at both protein and nucleotide levels based on proteome data in developmental strawberry fruits. (A) Three continually-declined polypeptides related to putative polyamine oxidase5 were annotated at protein expression levels around the onset of strawberry fruit ripening at the LG, Wt, IR, and PR stages in the National Center of Biotechnology Information (NCBI) web site (<https://blast.ncbi.nlm.nih.gov/Blast.cgi>). (B) Real-time PCR analysis of *FaFaPAO5* in SG, LG, DG, Wt, IR, PR, and FR fruits. . The *actin* expression level was used as an internal control. Error bars represent standard errors (*n* = 3). Columns with different letters (a-f) indicate statistically significant difference (p < 0.05) when the data are performed by variance analysis followed by Duncan’s multiple range tests. SG: small green; LG: large green; DG: de-greening; Wt: white; IR: initial red; PR: Partial red; FR: full red.

**
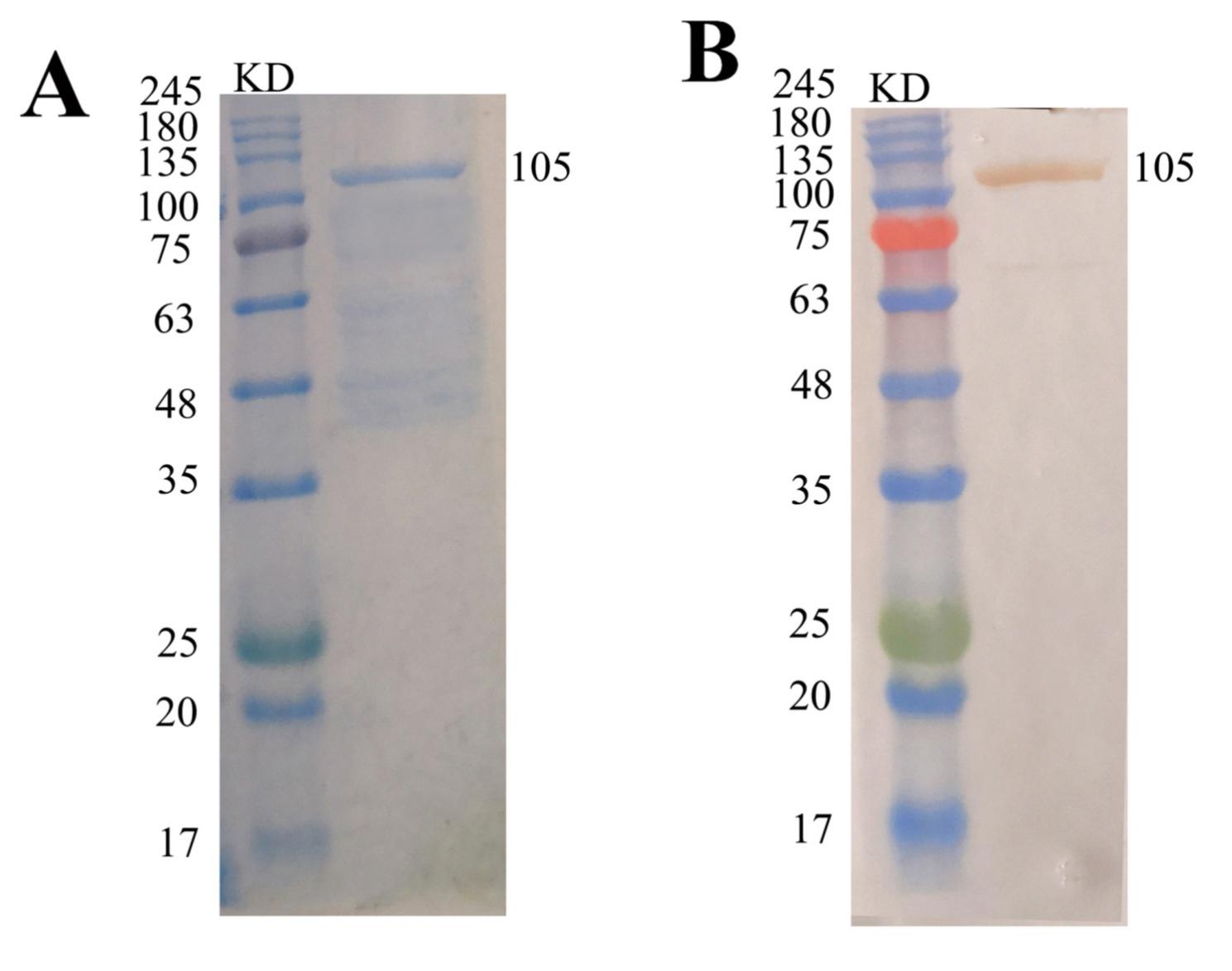
**

**Supplemental Figure S2.** SDS-PAGE and western blotting analysis of purified recombinant protein FaPAO5 gained from *E. coli* cells. (A) SDS-PAGE analysis of recombinant FaPAO5. (B) Western blotting analysis of recombinant FaPAO5 by an anti-His tag antibody using a one-step western kit HRP.


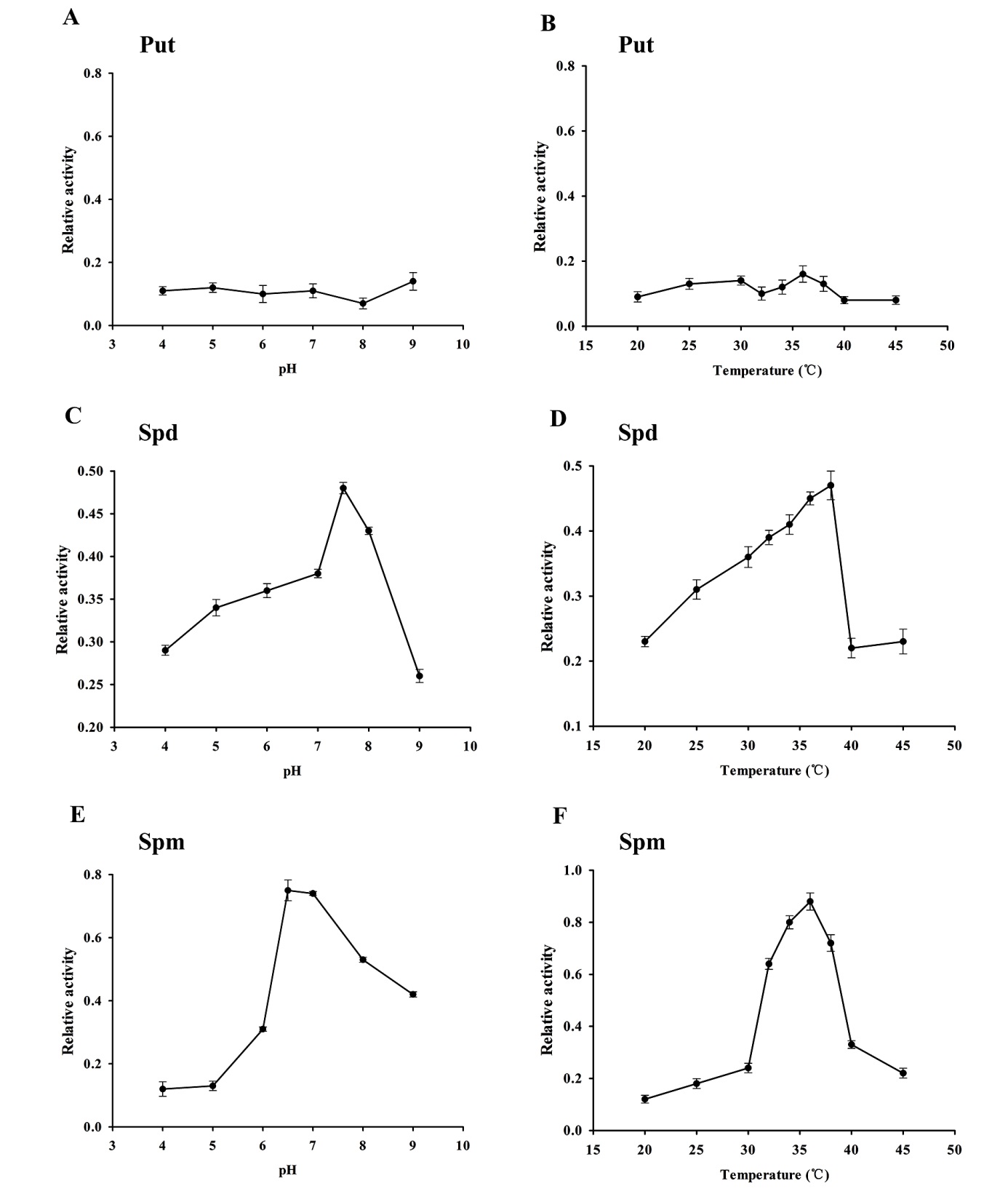


**Supplemental Figure S3.** Effects of pH and temperature on FaPAO5-polyamine reaction by H_2_O_2_ production. (A-B) FaPAO5-Put reactions. (C-D) FaPAO5-Spd reactions. (E-F) FaPAO5-Spm reactions. Error bars represent standard errors (*n* = 3).


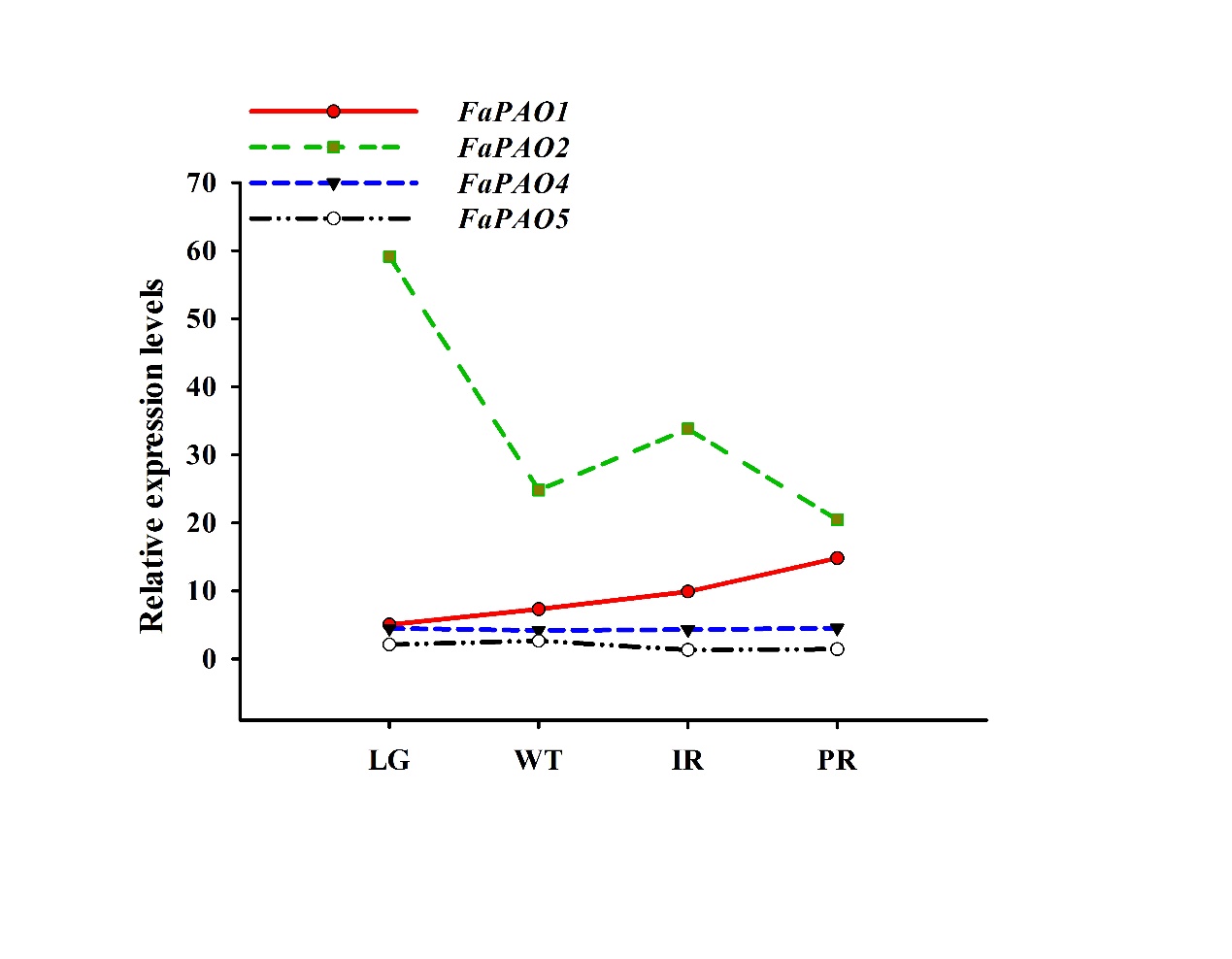


**Supplemental Figure S4.** Expression patter of PAO family based on transcriptome data in developmental strawberry fruits. LG: large green; WT: white; IR: initial red; PR: Partial red. (Bioproject accession: PRJNA438551)


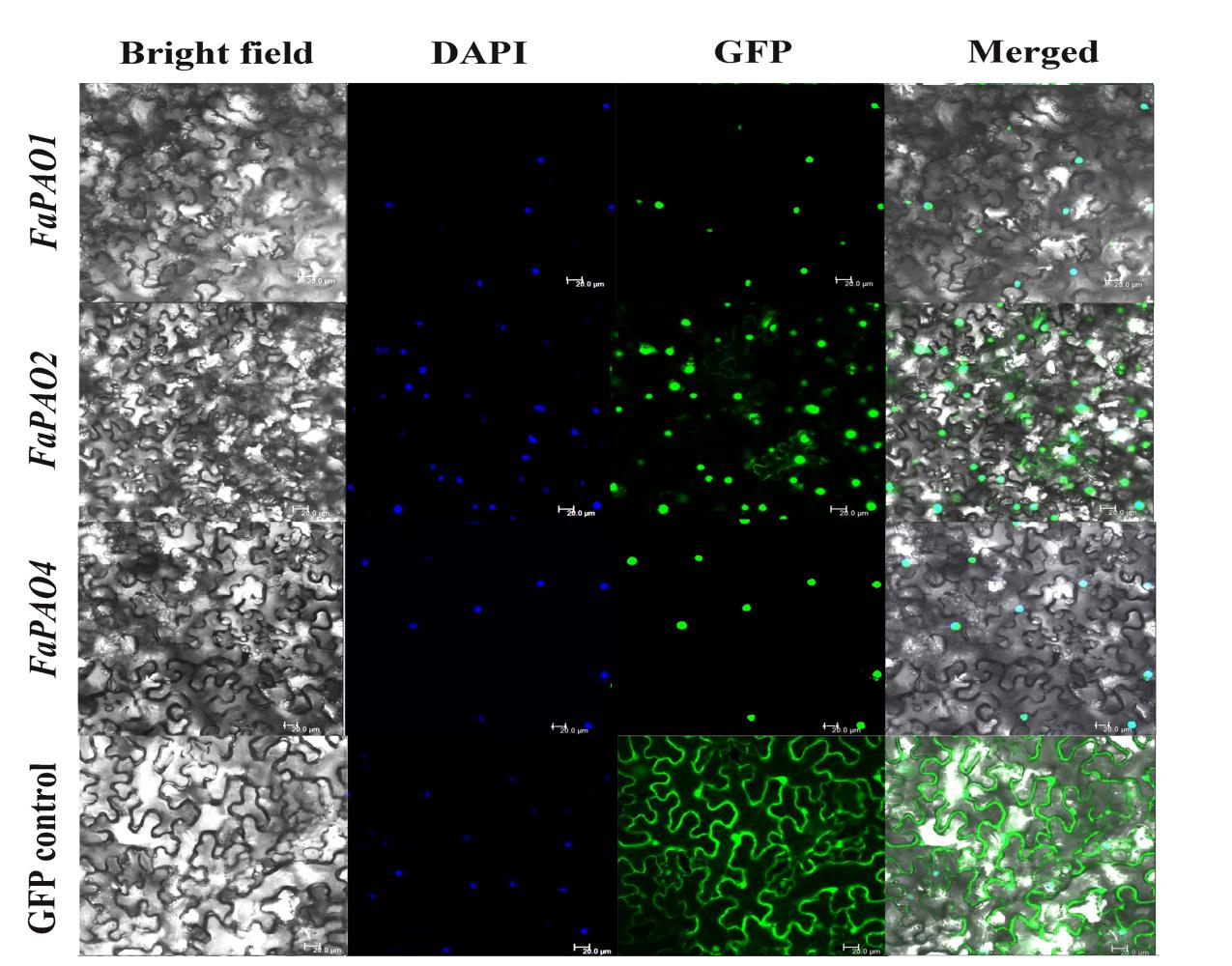


**Supplemental Figure S5.** Subcellular localizations of PAO1、PAO2 andPAO4. Localization analysis was performed to use a green fluorescent protein (GFP)-pCAMBIA1300-ProSuper–FaPAO1-4 fusion protein (GFP-FaPAO5) in *Nicotiana tabacum* leaves; and the GFP:pCAMBIA1300-ProSuper (1300-GFP) was used as control. Fluorescence images were obtained by confocal-laser scanning microscope. The nuclei stained by 4′,6′-diamidino-2-phenylindole (DAPI) appear in blue. Scale bar = 20 μm.


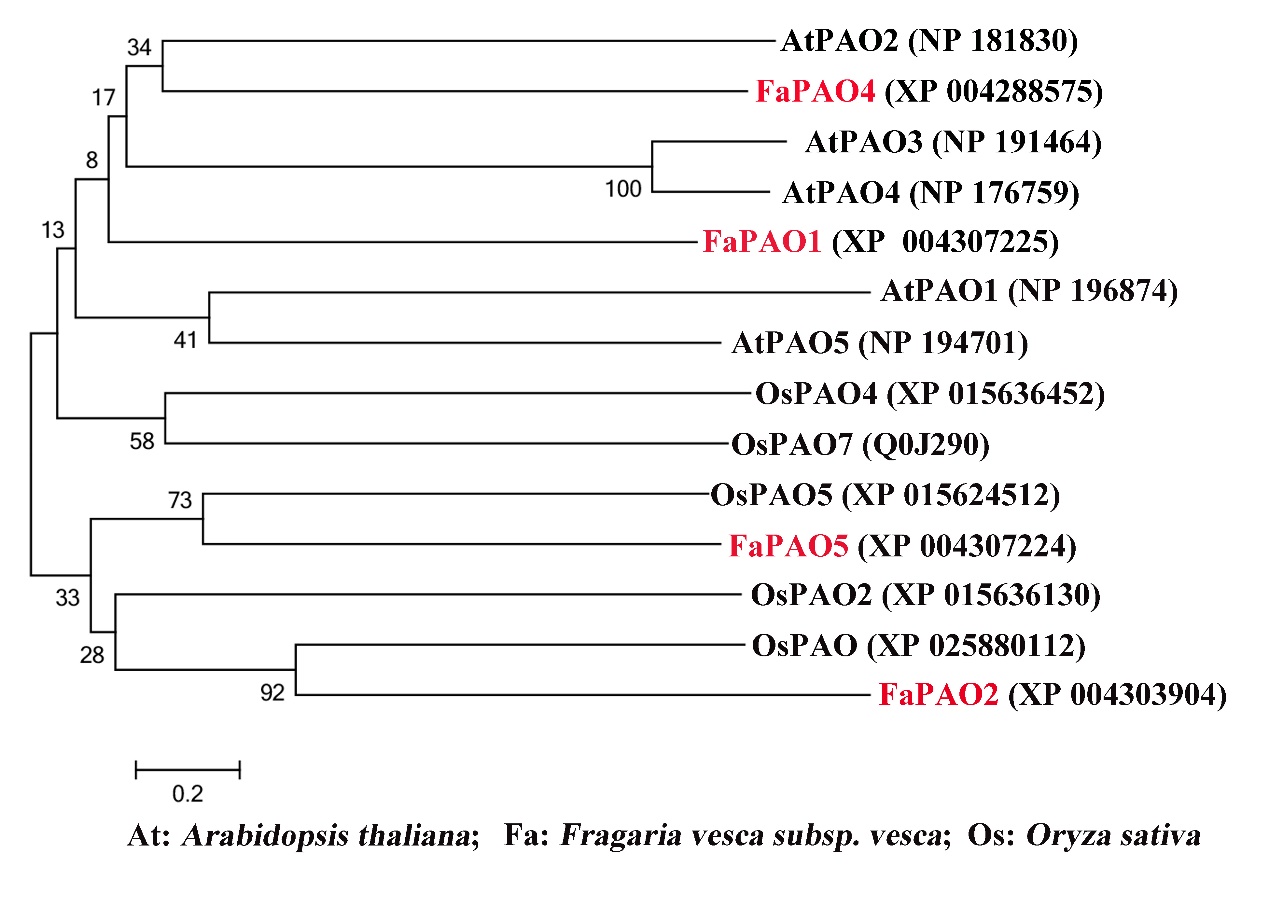


**Supplemental Figure S6.** Phylogenetic analysis of PAOs in different plants based on amino acid sequences. The phylogenetic tree of PAOs were independently built with MEGA5.1 using the bootstrap method. The reliability levels of internal branches were assessed using the bootstrap test (1,000 replicates).


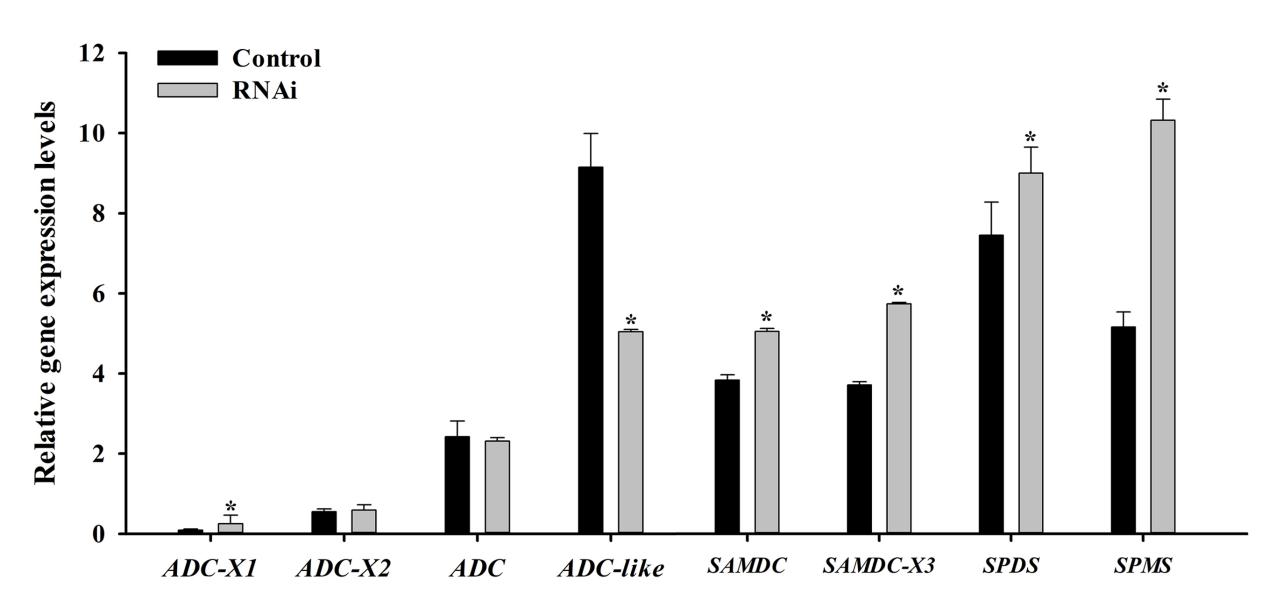


**Supplemental Figure S7.** The mRNA expression level of ADC, ODC, SAMDC, SPDS, SPMS family based on transcriptome data in PAO5-RNAi strawberry


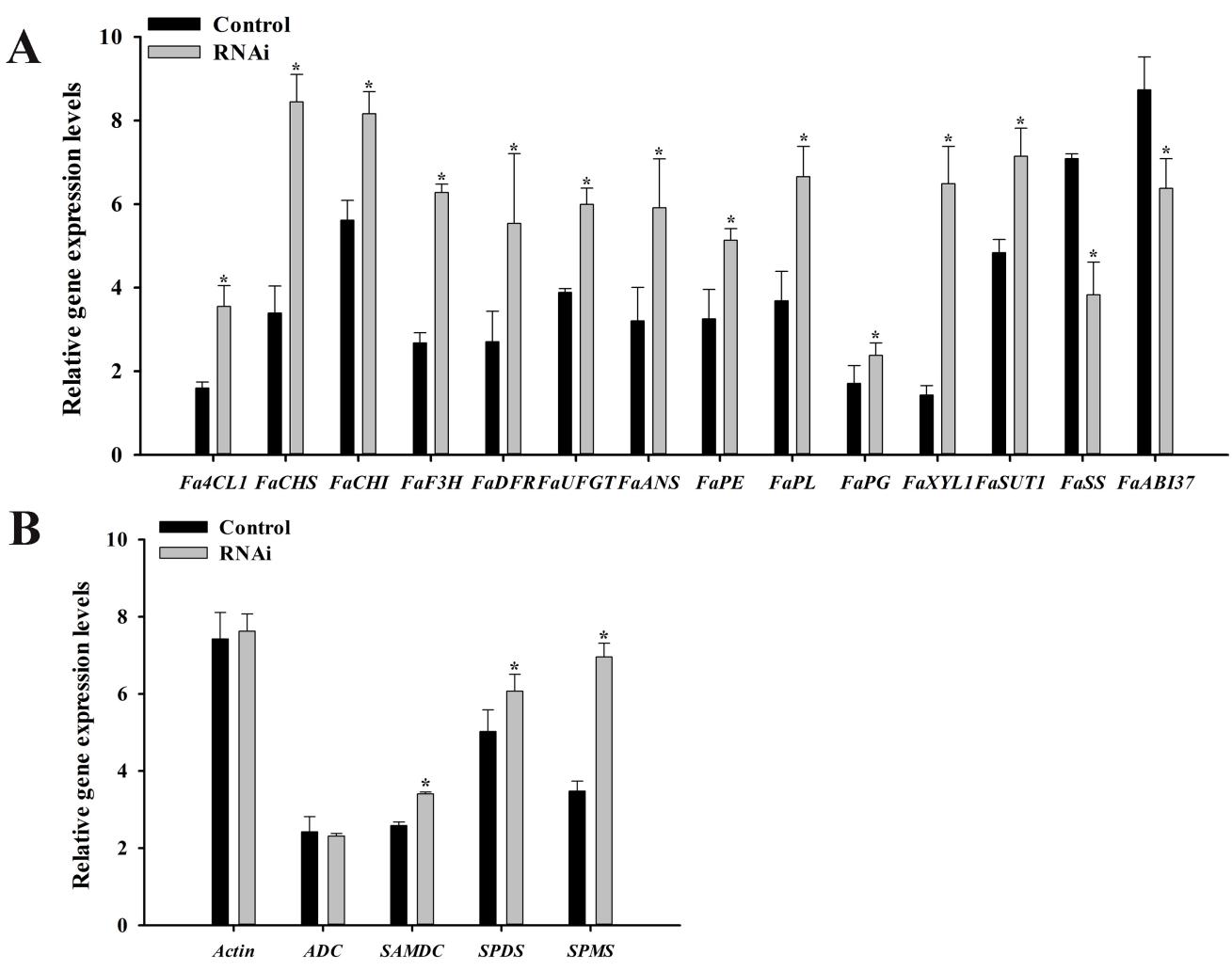


**Supplemental Figure S8.** Expressions of ripening and polyamine related genes in the based on transcriptome data in PAO5-RNAi strawberry.

(A) Expression levels ripening-related genes.

(B) Expression levels polyamine-related genes.
